# Regeneration of Thyroid Glands in the Spleen Restores Homeostasis in Thyroidectomy Mice

**DOI:** 10.1002/advs.202305913

**Published:** 2023-12-07

**Authors:** Xue‐Jiao Tian, Zhi‐Jie Yin, Zhen‐Jiang Li, Zhen‐Zhen Wang, Zhen Xing, Chun‐Yan Liu, Lin‐Tao Wang, Chun‐Ming Wang, Jun‐Feng Zhang, Lei Dong

**Affiliations:** ^1^ State Key Laboratory of Pharmaceutical Biotechnology School of Life Sciences Nanjing University Nanjing Jiangsu 210023 China; ^2^ NJU Xishan Institute of Applied Biotechnology Xishan District Wuxi Jiangsu 214101 China; ^3^ State Key Laboratory of Quality Research in Chinese Medicine Institute of Chinese Medical Sciences University of Macau Taipa Macau SAR 999078 China; ^4^ National Resource Center for Mutant Mice Nanjing 210023 China; ^5^ Chemistry and Biomedicine Innovative Center Nanjing University Nanjing Jiangsu 210023 China

**Keywords:** homeostasis restoration, spleen, thyroid regeneration

## Abstract

Surgical removal of the thyroid gland (TG) for treating thyroid disorders leaves the patients on lifelong hormone replacement that partially compensates the physiological needs, but regenerating TG is challenging. Here, an approach is reported to regenerate TG within the spleen for fully restoring the thyroid's functions in mice, by transplanting thyroid tissue blocks to the spleen. Within 48 h, the transplanted tissue efficiently revascularizes, forming thyroid follicles similar to the native gland after 4 weeks. Structurally, the ectopically generated thyroid integrates with the surrounding splenic tissue while maintaining its integrity, separate from the lymphatic tissue. Functionally, it fully restores the native functions of the TG in hormone regulation in response to physiological stimuli, outperforming the established method of oral levothyroxine therapy in maintaining systemic homeostasis. The study demonstrates the full restoration of thyroid functions post‐thyroidectomy by intrasplenic TG regeneration, providing fresh insights for designing novel therapies for thyroid‐related disorders.

## Graphical abstract

The intrasplenic transplanted thyroid tissue blocks undergo angio‐follicular unit (AFU) reconstruction, epithelial and follicular regeneration, and finally, a regenerated thyroid gland that is independent and seamlessly integrated with the spleen taking advantage of the abundant vascularity of the spleen and the diversity of growth factors. The regenerated intrasplenic thyroid achieves precise hormone release and synthesis, fully restoring all thyroid functions.

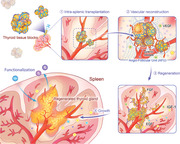

## 1 Introduction

Thyroid cancer remains a significant global health concern, with 500 000 new cases reported annually.^[^ ^1^ ^]^ Its primary mode of treatment, thyroidectomy (the surgical removal of the thyroid gland), leaves the patients on life‐long replacement therapy, typically by oral administration of levothyroxine sodium (L‐T_4_).^[^ ^2^ ^]^ This therapy manages to sustain some basic activities of thyroid hormones (THs) but fails to restore the vital functions of an intact thyroid, e.g. responsiveness and feedback regulation, leading to various disorders and complications.^[^ ^3^ ^]^ Also, clinical evidence suggests that THs deficiency increases the risks of hypertension,^[^ ^4^ ^]^ cardiac dysfunctions,^[^ ^5^ ^]^ and other metabolic or mental health conditions.^[^ ^3^, ^6^ ^]^

Thyroid tissue transplantation has been a viable solution for restoring thyroid function post‐thyroidectomy, particularly in cases of early‐stage thyroid cancer.^[^ ^7^ ^]^ The harvested thyroid, upon removal of the small portion of cancerous cells therein *ex vivo*, can be implanted back into the patient's body to restore thyroid functions.^[^ ^8^ ^]^ This approach could mitigate the drawbacks associated with oral thyroid hormone therapy. For allogeneic or xenogeneic transplantations, it requires a lifetime of immunosuppressive drugs, which outweighs the benefits. Therefore, clinical practices generally prefer autologous thyroid tissue transplantation. However, it is challenging to reconstruct thyroid tissue in situ in patients, due to surgical constraints on the circulatory system and the vascular structure of the thyroid itself.^[^ ^9^ ^]^ Aimed at these obstacles, ectopic transplantation might be another promising strategy. The thyroid is a typical endocrine organ regulated by physiological signals within the bloodstream, which can theoretically be successfully reconstructed ectopically without affecting its function. Since 1967,^[^ ^10^ ^]^ researchers conducted thyroid autotransplantation experiments in various animals, including guinea pigs,^[^ ^11^ ^]^ rats,^[^ ^12^ ^]^ rabbits,^[^ ^13^ ^]^ and dogs,^[^ ^14^ ^]^ and initiated preliminary technical exploration in human clinical trials. These studies mainly chose muscles or subcutaneous sites for transplantation.^[^ ^15^ ^]^ Nonetheless, these regenerative strategies did not yet yield satisfactory outcomes. The limited success could impute the poor integration with the host tissue, low survival rates, slow functional recovery, and others.^[^ ^15^, ^16^ ^]^

The key to restoring thyroid functions is maintaining the structure of thyroid follicles, which are the unique characteristics of this gland and responsible for the core functions of hormone synthesis and storage.^[^ ^17^ ^]^ They rapidly release hormones in response to thyroid‐stimulating signals and constitute the thyroid's feedback control system. However, two major requirements need addressing for maintaining the integrity of the thyroid follicles for transplantation. The first is a venue with enough space; the muscle has a dense structure, which poses significant challenges to thyroid transplantation, as tested before; the second is an established blood supply system.^[^ ^18^ ^]^ In the thyroid organ, the angio‐follicular unit (AFU),^[^ ^19^ ^]^ comprising a basket‐like network of capillaries covering the thyroid follicles, plays a vital role in rapid hormone response. For thyroid transplantation, the recipient tissue should provide a microenvironment supporting neovascularization and the establishment of the AFU. Nevertheless, these essential requirements remain unfulfilled by existing technologies.

Here, we propose a new method to grow thyroids in the spleen, by utilizing the unique characteristics of this organ. Its loose structure and abundant vasculature offer space and blood supply for thyroid growth,^[^ ^20^ ^]^ and the high storage of multiple growth factors (GFs), including insulin‐like growth factor 1 (IGF‐1), and vascular endothelial growth factor (VEGF), provide extra advantages for facilitating transplant growth and AFU formation. Our recent success in regenerating functional liver tissue within the spleen demonstrated the feasibility and safety of regenerating a different tissue in the spleen;^[^ ^21^ ^]^ given the smaller size of the thyroid gland compared to the liver, growing thyroids perhaps has minimal impacts on the original splenic functions. To validate this concept, we transplanted thyroid tissue blocks into the spleens of hypothyroid mice and monitored the regeneration process over time. By properly controlling the sizes of tissue blocks, the survival of the transplanted thyroid tissue within the spleen could be fairly high. Our results demonstrated the complete restoration of thyroid functions, as evidenced by normal thyroid hormone levels and the morphological characteristics of the regenerated gland.

## 2 Results

### 2.1 Transplantation of Thyroid Tissue Grafts into Mouse Spleens

**Figure 1:**
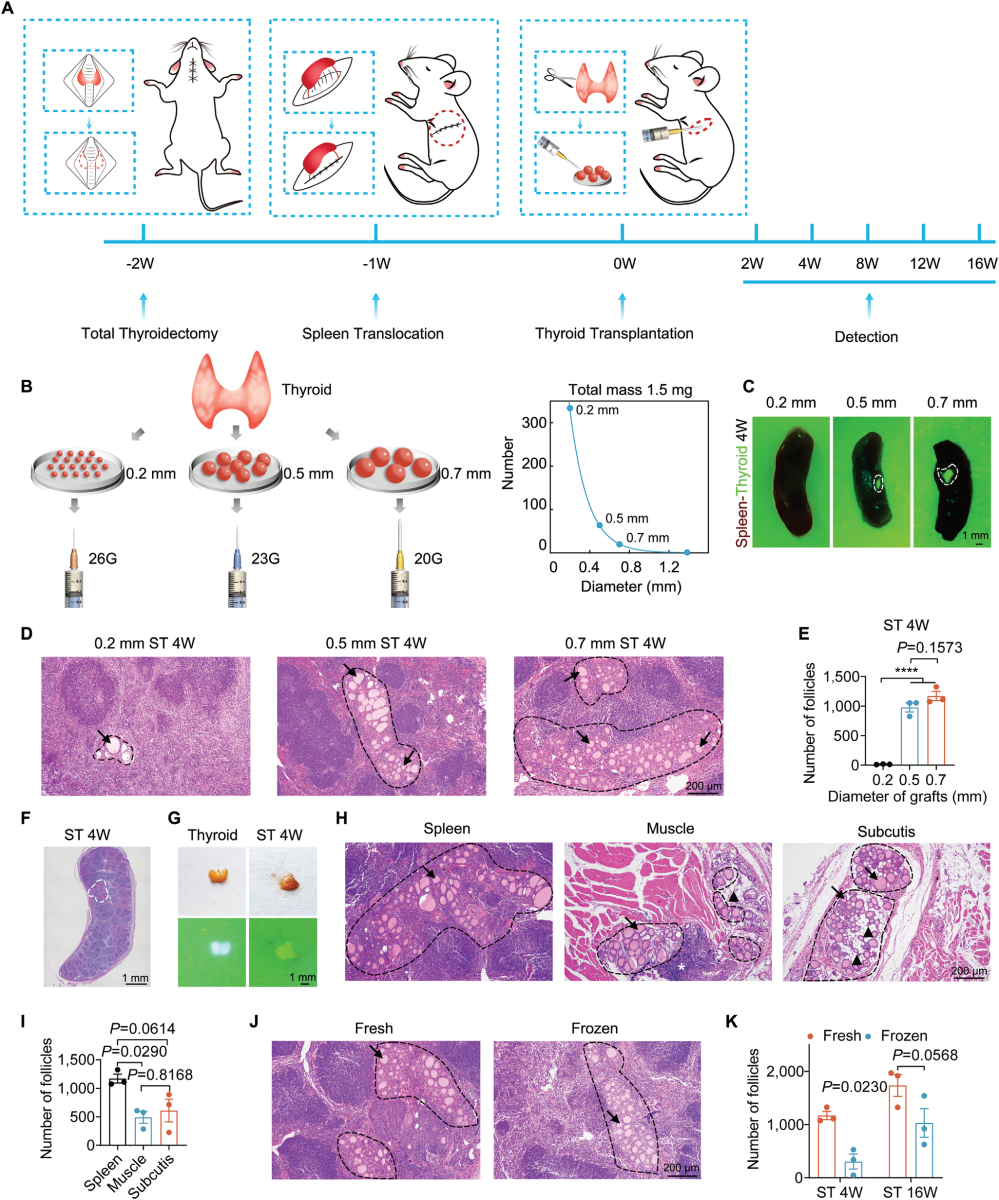
Thyroid transplantation into translocated mouse spleens. A) Surgical procedure schematic illustrating thyroidectomy, spleen translocation, and intra‐splenic thyroid transplantation. B) Schematic diagram depicting the preparation of thyroid tissue blocks and the correlation between the number of tissue blocks and their sizes. GFP‐labeled tissue blocks of varying sizes were transplanted into spleens for 4 weeks. C) Observation of the transplanted tissue blocks 4 weeks after transplantation under a 488 laser lamp and filter. Thyroid graft sites are highlighted within white dashed curves (‐ – ‐). D) H&E staining of tissue sections showing intra‐splenic thyroid tissues (ST) 4 weeks after transplantation. The black arrows (**→**) indicate the follicular structure, and the black dashed curves (‐ – ‐) outline the ST tissue. E) Quantification of follicles in the intra‐splenic thyroid 4 weeks after transplantation. *n* = 3 biological replicates. F) PAS staining of a whole‐spleen section containing intra‐splenic thyroid tissues (indicated by white dashed curves (‐ – ‐)) 4 weeks after transplantation. G) Surgical separation of regenerated thyroid ST (developed from transplanted tissue blocks with 0.7 mm diameters) from the spleen (bright field and laser 488). H) H&E staining analysis comparing intra‐splenic, intra‐muscle, and subcutaneously transplanted thyroid tissues 4 weeks after the transplantation. Black dashed curves (‐ – ‐) outline the ST tissue, black arrows (**→**) indicate typical thyroid follicular structures, black triangles (**▲**) represent disappeared follicles, and white asterisks (*) indicate necrotic tissues. I) Calculation of thyroid follicle numbers in the spleen, muscle, and subcutis, respectively, 4 weeks after the transplantation. *n* = 3 biological replicates. J) H&E staining comparison between cryopreserved and freshly transplanted thyroid tissues at 4 weeks post‐transplantation. Thyroid follicles were labeled with black arrows (**→**), and the black dashed curves (‐ – ‐) outline the ST tissue. K) Quantification of thyroid follicles in ST counted at weeks 4 and 16. *n* = 3 biological replicates. Data presented as means ± SEM. Statistical analyses were performed using one‐way ANOVA followed by Tukey's multiple comparisons tests for (E,I) and two‐way ANOVA followed by *Tukey's* multiple comparisons tests for (K). ****P ≤ 0.0001. PAS, periodic acid‐Schiff; ST, intra‐splenic thyroid; ANOVA, analysis of variance.

Our previous studies have demonstrated the regenerative capacity and functionality of transplanted liver tissue within the spleen.^[^ ^21^ ^]^ In this study, we aimed to explore the potential for intrasplenic thyroid regeneration by optimizing the processing protocol for donor tissues and the transplantation procedure. For this purpose, we utilized mouse models with thyroidectomy and spleen translocation as experimental subjects to assess the efficacy of our approach (**Figure** **1**A). To simulate autologous tissue transplantation performed in clinical settings, the donor mice shared the same genotype as the recipients. Histological and physiological examinations were conducted at 2, 4, 8, 12, and 16 weeks post‐transplantation. The processing of donor thyroid tissue involved the collection of the donor mouse's thyroid gland, followed by washing, cutting, and dispersing it in a hyaluronic acid solution before direct injection into the spleen (Figure 1A). The total mass of transplanted tissue was standardized to 1.5 mg per mouse. Based on our previous studies, the optimization of the protocol primarily focused on the effects of varying tissue graft diameters (0.2 mm, 0.5 mm or 0.7 mm) (Figure 1B, Figure S1A, Supporting Information) on regeneration outcomes after 4 weeks of transplantation. To facilitate visualization, we employed green fluorescent protein (GFP) transgenic mice with GFP‐labeled thyroid tissue as donors. The regenerated GFP‐labeled thyroid tissue 4 weeks after transplantation could be directly observed using a 488 nm laser lamp and filter (Figure 1C). Our results indicated robust survival of larger grafts (0.5 mm or 0.7 mm) in the spleen, whereas tissue blocks of 0.2 mm completely disappeared from the transplantation site. Subsequent histological analysis employing hematoxylin‐eosin (H&E) staining (Figure 1D) and follicle counting (Figure 1E) revealed the presence of a typical thyroid histological structure characterized by multiple integrated follicles within the tissue graft. Quantitative analysis demonstrated the presence of more than 1,000 follicles in a single tissue block. Considering the size and structure of the mouse spleen, particularly its thickness, we determined that grafts with a diameter of 0.7 mm represented the largest size that could be safely transplanted into the spleen without inducing bleeding or other safety concerns. Consequently, we selected 0.7 mm tissue blocks for all subsequent experiments. In representative transplantation utilizing 0.7 mm tissue blocks and allowing for 4 weeks of growth within the spleen, glycoprotein‐specific periodic acid‐Schiff (PAS) staining (Figure 1F) revealed a uniform regenerated tissue within the spleen. Surprisingly, the regenerated thyroid tissue in the spleen could be isolated from the surrounding spleen tissue, displaying a comparable volume and gross morphology to that of the native thyroid (Figure 1G). Surprisingly, though we did not intentionally transplant the parathyroid tissue, through immunohistochemical labeling of parathyroid hormone (PTH) and calcium‐sensitive receptor (CaSR), we did observe the presence of surviving parathyroid glands in some of the splenic tissues (Figure S1B, Supporting Information).

Subsequently, we conducted a comparative analysis between the regeneration outcomes of thyroid tissues transplanted in the spleen and those transplanted in muscle or subcutaneous sites. As depicted in Figure 1H, the intramuscular or subcutaneous transplantation of thyroid tissues still existed in the transplanting sites after 4 weeks of surgery (Figure 1I), however, extensive tissue necrosis, follicular detachment, and a reduced number of follicles were observed. Considering the potential utilization of cryopreservation in clinical practice, we examined whether thyroid tissues subjected to a typical freeze‐thaw process could still regenerate within the spleen. The results presented in Figure 1J,K revealed that frozen thyroid tissue exhibited lower regeneration efficiency compared to fresh tissue; however, after 16 weeks within the spleen, it attained a similar size to that of fresh tissue and restored comparable serum hormone levels (Figure S1C,D, Supporting Information) when cryopreserved tissues were employed.

Last, we investigated the impact of thyroid tissue transplantation and growth on splenic function. Firstly, after translocating the spleen from the peritoneal cavity to the subcutaneous area (Figure S1E, Supporting Information), we observed no significant changes in the appearance (Figure S1F, Supporting Information) and weight (Figure S1G, Supporting Information) of the spleen hosting the thyroid tissue. Second, upon scanning a whole spleen section, it became evident that the thyroid tissue occupied a relatively limited space within the spleen, with the regenerated tissue weighing approximately 2–3 mg, which was considerably small compared to the overall spleen weight of around 80 mg. Thirdly, we conducted cell type analysis in the spleen by counting the percentage of different cell types (Figure S1H,I, Supporting Information) and estimating the immune cell composition (Figure S2A, Supporting Information) and immune infiltration score (Figure S2B, Supporting Information) using ImmuCC, a mouse‐specific algorithm capable of inferring the ratio of 25 immune cells.^[^ ^22^ ^]^ We further employed principal component analysis (PCA) analysis (Figure S2C, Supporting Information) and Spearman's clustering analysis (Figure S2D, Supporting Information) based on the spleen expression profile to assess changes. Notably, no significant alterations were observed in the spleen before and after transplantation by the Kyoto Encyclopedia of Genes and Genomes (KEGG) pathway analysis, except for the emergence of functions associated with thyroid hormones (Figure S2E, Supporting Information). Fourthly, a blood routine examination revealed no systemic pathological changes following the intrasplenic operation (Table S1, Supporting Information). Finally, we attempted thyroid transplantation using different genotypes of donor and recipient mice. Unfortunately, the grafts failed to survive beyond 10 days in the host due to an aggressive immune rejection reaction (Figure S2F–H, Supporting Information).

Collectively, our experimental findings provide evidence that autologous intrasplenic transplantation of thyroid tissue results in successful survival within the spleen without significantly affecting normal spleen functions.

### 2.2 Regeneration of Transplanted Thyroid Tissue in the Spleen

**Figure 2:**
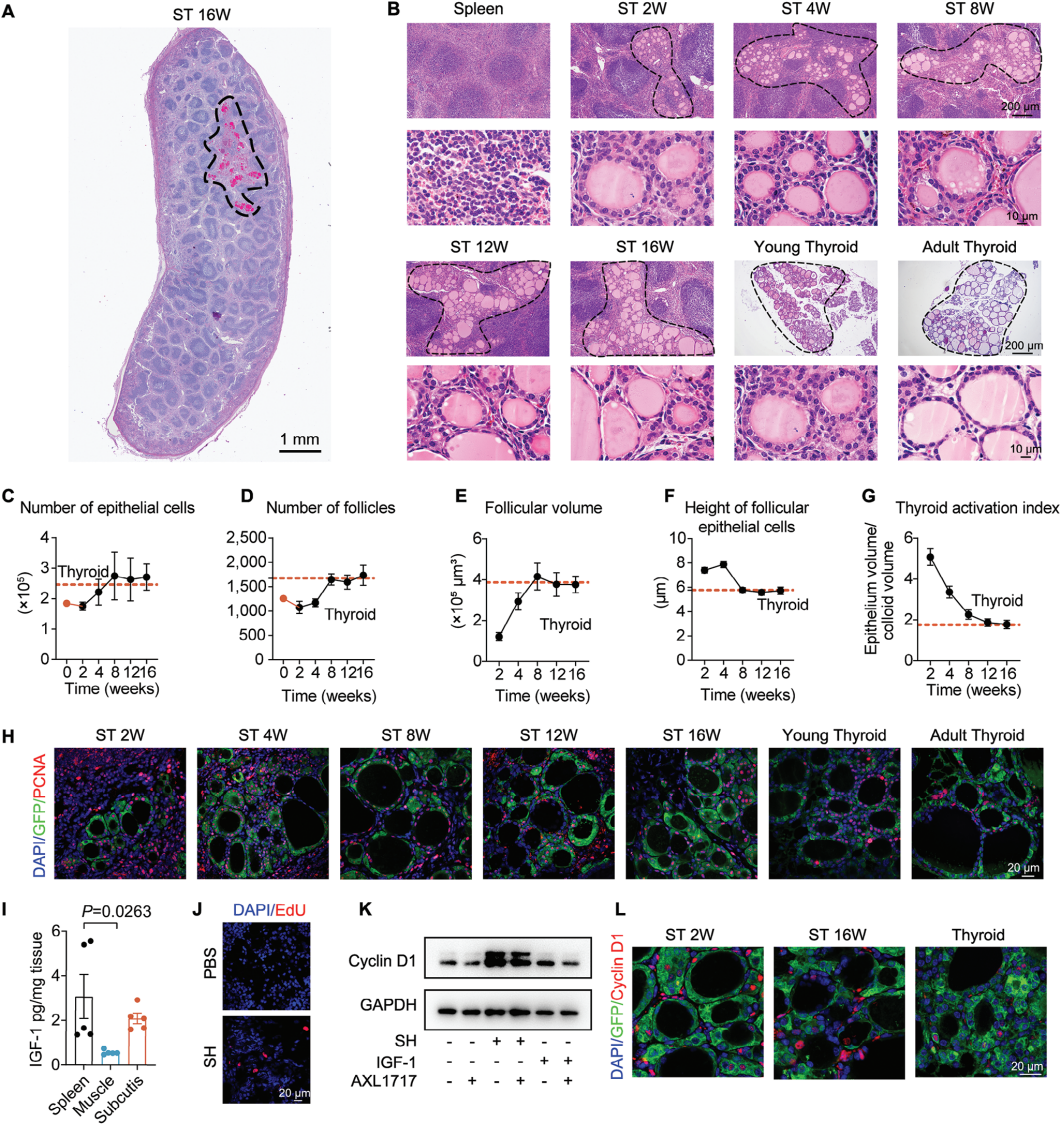
Regeneration of intra‐splenic transplanted thyroid tissue. A) PAS staining of a whole‐spleen section showing the regenerated thyroid tissue (ST) in the spleen 16 weeks post‐transplantation. The black dashed curves (‐ – ‐) outline the ST tissue. B) H&E staining analysis of the intra‐splenic regenerated thyroid tissue at weeks 2, 4, 8, 12, and 16. Upper panels show the thyroid tissue (ST) within the spleen, while lower panels provide detailed views of the regenerated thyroid follicles. The black dashed curves (‐ – ‐) outline the ST tissue. C) Weekly monitoring of the number of thyroid follicular epithelial cells in the intra‐splenic thyroid tissue for 16 consecutive weeks, with adult thyroid as control. The value for week 0 represents the calculated number of cells in the transplanted tissue blocks. *n* = 5 biological replicates. D) Weekly assessment of the number of thyroid follicles in the intrasplenic thyroid tissue for 16 weeks, with adult thyroid as control. The value for week 0 represents the calculated number of follicles in the transplanted tissue blocks. *n* = 3 biological replicates. E) Measurement of the volume of thyroid follicles, with adult thyroid as control. *n* = 40. F) Determination of the height of thyroid follicular epithelial cells, with adult thyroid as control. *n* = 50. G) Calculation of the thyroid follicular activation index, with adult thyroid as control. *n* = 50. H) Evaluation of proliferative activity through PCNA immunofluorescence staining (co‐staining with GFP to identify thyroid tissue in GFP‐transgenic mice). I) Comparison of IGF‐1 levels in spleen, muscle, and subcutis. *n* = 5 biological replicates. J) The proliferative activity of thyroid tissue blocks after co‐incubation with spleen homogenate (SH) was determined by EdU staining. K) Western blotting analysis of the Cyclin D1 expression in thyroid tissues after co‐incubation with spleen homogenate (SH), IGF‐1, and the inhibitor of the IGF‐1 receptor (picropodophyllin, AXL1717). L) Immunofluorescence staining of Cyclin D1 expression in the intra‐splenic regenerated thyroid tissue at weeks 2 and 16. Data presented as means ± SEM. Statistical analyses were performed using one‐way ANOVA followed by Tukey's multiple comparisons tests for (I). PAS, periodic acid‐Schiff; ST, intra‐splenic thyroid; SH, spleen homogenate; EdU, 5‐ethynyl‐2′‐deoxyuridine; ANOVA, analysis of variance.

In our study, it is noteworthy that the transplanted thyroid tissue within the spleen exhibited unexpected outcomes compared to conventional transplantation methods, where tissue blocks typically undergo necrosis or decline due to inadequate blood supply before vascularization.^[^ ^23^ ^]^ Surprisingly, we observed that the thyroid tissues within the spleen, 16 weeks post‐transplantation, were either the same size or more prominent than the total volume of the transplanted thyroid tissue blocks (**Figure** **2**A).

To investigate this phenomenon, we conducted a 16 week monitoring of tissue blocks post‐implantation into the spleen, employing a series of histological and quantitative analyses. Both young and adult thyroids, obtained from 4‐week‐old and 6‐month‐old mice, respectively, served as controls (Figure 2B). Our focus encompassed the dynamics of epithelial cells and follicles, crucial functional units, along with the overall thyroid structure. Notably, these parameters exhibited an escalating trend from weeks 2 to 8 following an initial decline, ultimately reaching a peak and maintaining stability after 8 weeks (Figure 2C,D). This suggests the spleen's potential suitability for transplanted thyroid growth. Simultaneously, we undertook a detailed analysis of follicular volume and the height of follicular epithelial cells, as these metrics can reflect the functional status of thyroid follicles. In active thyroid follicles with heightened functionality, epithelial cells typically assumed a cuboidal shape, resulting in higher follicular height despite smaller follicular volume.^[^ ^24^ ^]^ This scenario was observed in young thyroids. Conversely, adult thyroids exhibited flattened epithelial cells, leading to lower follicular height despite larger follicular volume.^[^ ^25^ ^]^ Based on these observations, we distinguished the active state of the transplanted thyroid. Notably, the growth pattern of the thyroid in the spleen mirrored the development of an active young thyroid during weeks 2 to 8 (Figure 2E,F), transitioning to a state similar to that of the adult thyroid, characterized by maximum volume and stable epithelial cell heights after 8 weeks. Additionally, the thyroid activation index (epithelial cell volume/colloid volume ratio), an indicator of proliferation activity, remained elevated relative to the adult thyroid from week 2 to 8 post‐transplantation (Figure 2G).^[^ ^26^ ^]^ These findings suggest not only the survival but also the potential proliferation of tissue within the spleen. To validate this hypothesis, we conducted immunofluorescent staining for proliferating cell nuclear antigen (PCNA) and quantified the proliferation by analyzing the percentage of PCNA^+^/GFP^+^ cells (Figure 2H, Figure S3A, Supporting Information). The results demonstrated a comparable proliferation of thyroid blocks relative to young mice. Furthermore, to ascertain whether proliferation primarily stemmed from stromal endothelial cells or functional epithelial cells, we meticulously evaluated endothelial cells in the blocks using endomucin (EMCN), a representative endothelial marker. Co‐staining of EMCN and PCNA revealed rare cellular co‐localization, excluding the main contribution of endothelial cells and blood vessels to proliferation (Figure S3B, Supporting Information). Thus, active proliferation appears to be closely associated with epithelial cells, potentially facilitating functional reconstruction.

We endeavored to elucidate the underlying factors contributing to the distinctive proliferation capacity observed in thyroid tissues within the spleen. Initially, we compared the concentrations of well‐documented thyroid epithelial cell proliferation activators, such as IGF‐1,^[^ ^27^ ^]^ in the spleen, with those in muscle or subcutaneous tissue. Our results revealed significantly higher levels of IGF‐1 in the spleen (Figure 2I). Subsequent in vitro experiments utilizing spleen tissue homogenate (SH), comprising soluble components of IGF‐1, demonstrated their ability to enhance the proliferation of thyroid epithelial cells, as evidenced by the EdU staining assay (Figure 2J). Furthermore, the expression of Cyclin D1,^[^ ^28^ ^]^ a typical cellular indicator of proliferation and a downstream effector of IGF‐1 signaling, exhibited a significant elevation in thyroid tissue blocks following SH treatment (Figure 2K). The heightened expression of Cyclin D1 in the intrasplenic thyroid, relative to the natural thyroid, further supports the notion that the proliferative capacity of the thyroid in the spleen could be stimulated by IGF‐1 (Figure 2L; Figure S3C, Supporting Information). Additionally, the administration of an inhibitor of the IGF‐1 receptor (picropodophyllin, AXL1717) attenuated the proliferation effects in both in vitro culture tests (Figure 2K) and *in vivo* intra‐splenic transplantation (Figure S3D,E, Supporting Information). These findings suggest that the abundant presence of IGF‐1 in the spleen might play a pivotal role in the ectopic regeneration of thyroid tissue within the spleen.

### 2.3 Reconstruction of AFU and Integration of Regenerated Thyroid Tissue in the Spleen

**Figure 3:**
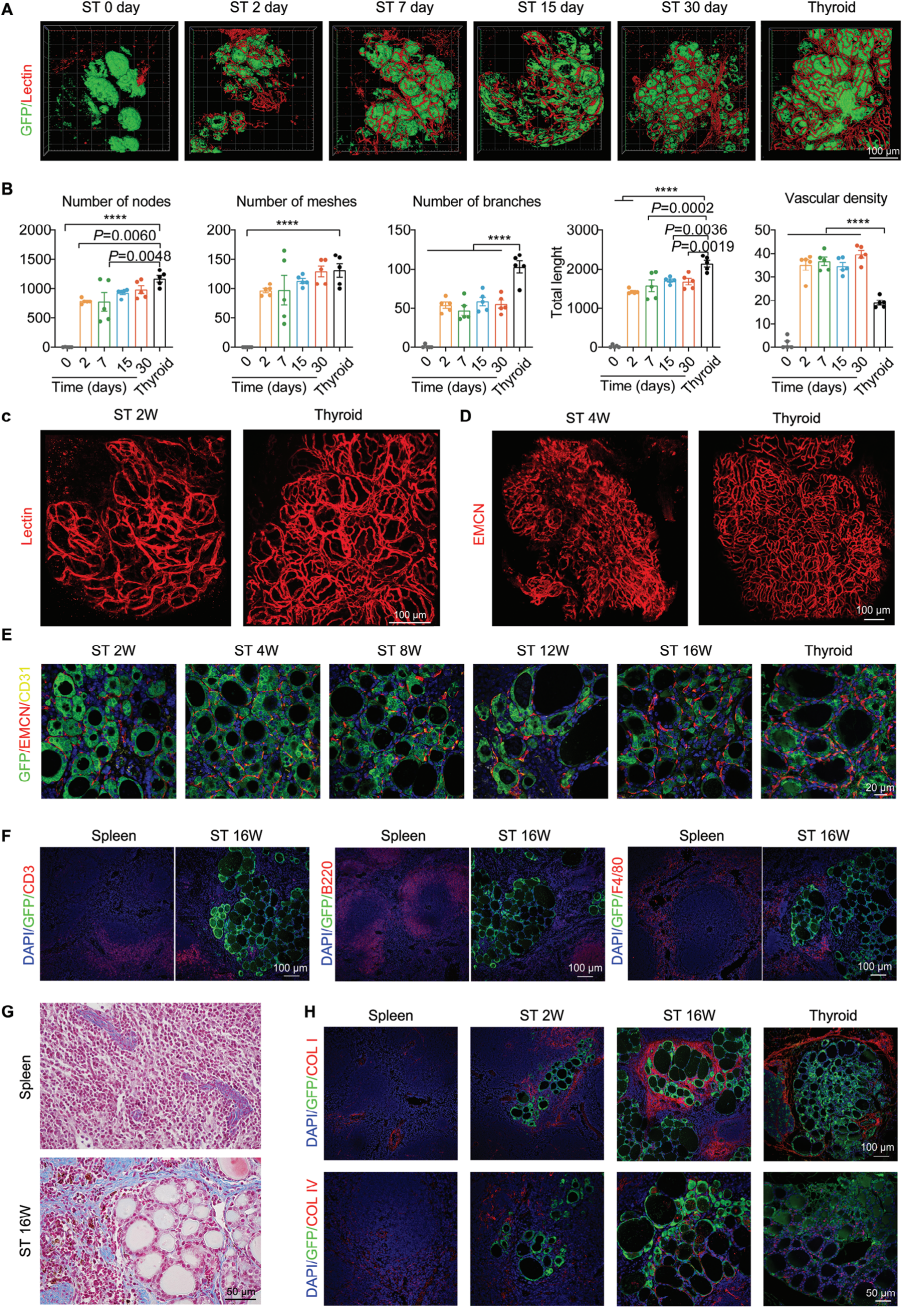
Reconstruction of AFU and integration of regenerated thyroid tissue with the spleen. A) Confocal 3D imaging of the vascular network (labeled with DyLight*649 Lycopersicon esculentum lectin) to monitor vascularization in the GFP‐labeled thyroid grafts post‐transplantation. B) Quantitative analysis of vessel nodes, vascular meshes, vascular branches, total vessel length, and vessel density based on imaging data. *n* = 5. C) 3D images of a lectin‐labeled angiogram of intrasplenic thyroid vessels at 2 weeks after transplantation, compared with the natural thyroid. D) Confocal 3D imaging of EMCN‐labeled blood vessels in ST blocks at 4 weeks after transplantation, comparing them with the native thyroid gland. E) Immunofluorescence staining of EMCN and CD31 in sections of the intra‐splenic thyroid at 2 to 16 weeks after transplantation. F) Immunofluorescence staining for T cells (CD3^+^), B cells (B220^+^), and macrophages (F4/80^+^) in spleen sections, comparing areas with ST integration to pure spleen tissue areas. G) Masson staining of the intra‐splenic thyroid, at 16 weeks after transplantation. H) Immunofluorescence staining for COL I and COL IV in the ST. Data presented as means ± SEM. Statistical analyses were performed using one‐way ANOVA followed by Tukey's multiple comparisons tests for (B). **** *P* ≤ 0.0001. ST, intra‐splenic thyroid; ANOVA, analysis of variance.

Efficient vascular reconstruction is crucial for the survival and regeneration of transplanted tissues, as it helps alleviate hypoxia and nutrient deprivation. The native thyroid gland possesses a distinctive angio‐follicular unit (AFU) structure,^[^ ^19^ ^]^ where a network of blood vessels surrounds the periphery of the follicles, enabling rapid response to hormones and other signals in the blood. In our study, we visualized the process of blood supply restoration to the transplanted tissue using DyLight 649‐labeled tomato lectin staining, which specifically labels live blood vessels. The confocal 3D reconstruction revealed the restoration of the thyroid gland's typical morphology and the development of blood vessels covering the tissue blocks. As shown in **Figure** **3**A, the blood supply to the transplanted tissue blocks recovered rapidly within 2 d post‐transplantation. We continued to monitor the revascularization process for 30 d and observed significant improvements in the number of vascular nodes, vascular meshes, vascular branches, total vessel length and vessel density (Figure 3B). Subsequently, a well‐formed basket‐liked AFU structure, highly resembling to the natural thyroid, was reconstructed in the intrasplenic thyroid gland, as shown with 3D angiography performed by lectin injection (Figure 3C) and immunofluorescence staining of endomucin (EMCN) in the thyroid tissue isolated from the spleen (Figure 3D). Immunofluorescent staining of blood vessel endothelial cell markers, EMCN and platelet endothelial cell adhesion molecule‐1 (CD31), further confirmed the formation of AFUs in the regenerated thyroid glands (Figure 3E).

As the native thyroid gland is an independent organ with no direct contact with other tissues, it was important to investigate whether the intra‐splenic regenerated thyroid organ maintained its independence or became mixed with the surrounding spleen tissue. As mentioned earlier, the regenerated thyroid tissue could be completely separated from the spleen (Figure 2A), indicating its independent existence despite growing within the spleen. To further examine this, we performed immunofluorescent staining of major splenic cell types (CD3^+^ for T cells, B220^+^ for B cells and F4/80^+^ for macrophages) in spleen sections containing thyroid tissues 16 weeks post‐transplantation. Minimal infiltration of splenic cells into the thyroid tissue was observed, and follicles were found to be clustered together (Figure 3F; Figure S4A–C, Supporting Information). These observations confirmed that the regenerated thyroid tissue within the spleen was physically separated from the surrounding splenic tissues. Furthermore, Masson staining (Figure 3G) and immunostaining of collagen markers (COL I, COL IV) (Figure 3H; Figure S4D,E, Supporting Information) for collagen revealed a membrane‐like structure rich in collagen surrounding the thyroid tissue in the spleen. This structure was presumed to originate from the co‐transplanted thyroid gland capsule, which maintained the integrity of the regenerated thyroid glands in the spleen after their own regeneration in the splenic environment.

Taken together, our findings demonstrate that the transplanted thyroid tissue efficiently reconstructs its circulatory system within the spleen. Although the regenerated thyroid glands integrate well with the spleen tissue, they possess their own isolated tissue space without infiltration of splenic cells or direct contact with the surrounding spleen environment. This suggests that these thyroid glands are capable of functioning normally as endocrine organs.

### 2.4 The Function of Regenerated Thyroid Tissue in the Spleen

**Figure 4:**
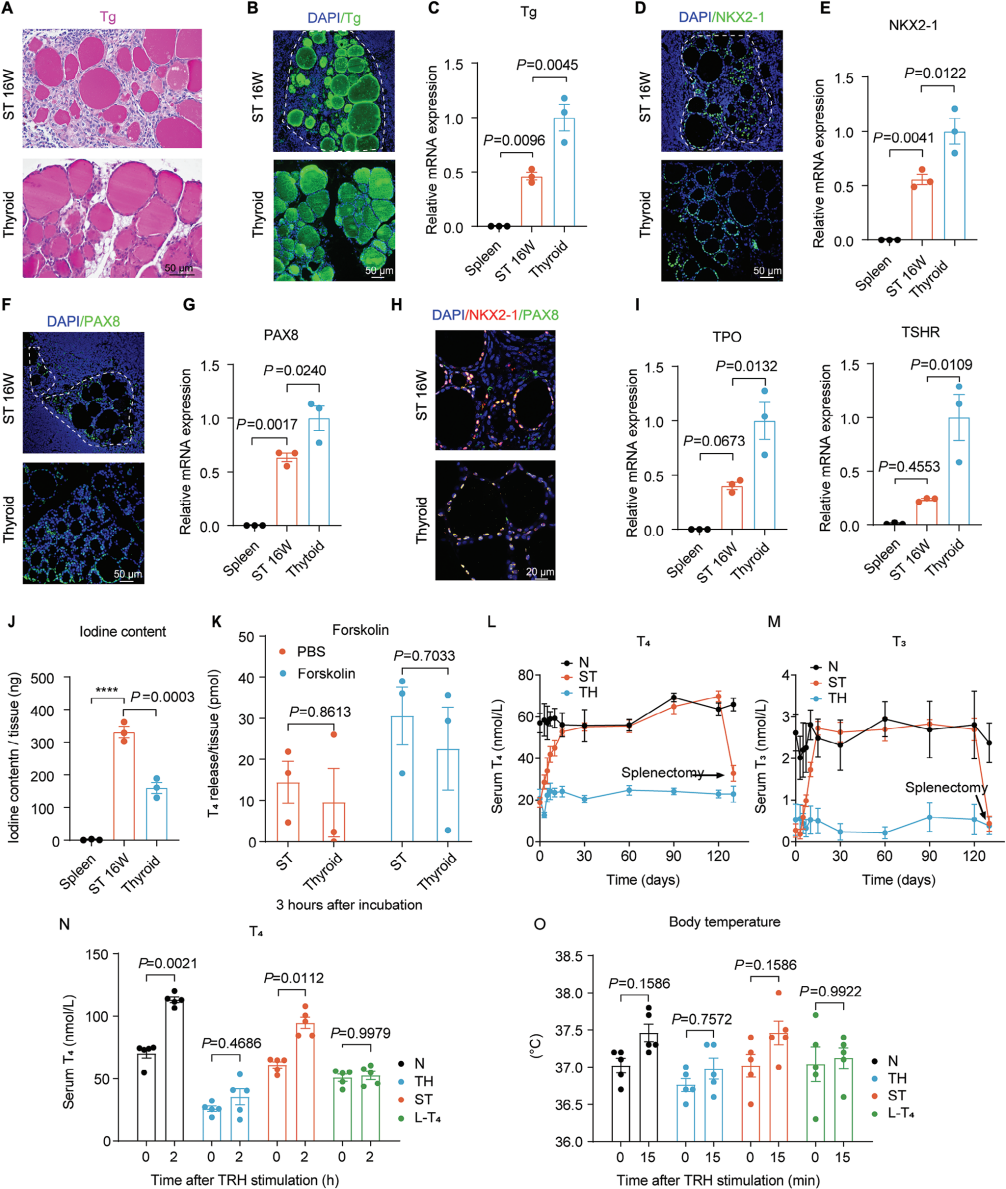
Function of the regenerated thyroid tissue in the spleen. A) PAS staining of thyroglobulin in the follicles of the intra‐splenic thyroid (ST) and native thyroid tissue. B–G) Immunofluorescence staining and relative mRNA expression for thyroid‐specific markers B,C) Tg. D,E) NKX2‐1 and F,G) PAX8. *n* = 3 biological replicates. The white dashed curves (‐ – ‐) outline the ST tissue. H) Immunofluorescence staining for the colocalization of NKX2‐1 and PAX8 in intrasplenic thyroid tissue. I) Relative mRNA expression of thyroid functional genes (*TPO* and *TSHR*) was measured by qRT‐PCR in the ST, with native spleen and thyroid tissues as control. *n* = 3 biological replicates. J) Iodine content in the ST, native thyroids, and spleens. *n* = 3 biological replicates. K) *Ex vivo* forskolin‐stimulated thyroid hormone (T_4_) release test. *n* = 3 biological replicates. L,M) Changes in serum L) T_4_ and M) T_3_ levels were measured in ST mice and following splenectomy surgery performed 130 d after thyroid tissue transplantation, with N mice as the control. *n* = 5 biological replicates. N,O) TRH simulations were performed on ST mice, and changes in serum N) T_4_ and O) body temperature were measured, with N mice, TH mice, and L‐T_4_ mice as controls. *n* = 5 biological replicates. The mice were divided into four groups for (L–O): N mice (normal healthy mice), ST mice (hypothyroid mice receiving intra‐splenic thyroid regeneration treatment), TH mice (mice with total thyroidectomy), and L‐T_4_ mice (hypothyroid mice receiving oral L‐T_4_ treatment). Data presented as means ± SEM. Statistical analyses were performed using one‐way ANOVA followed by Tukey's multiple comparisons tests for (C,E,G,I,J), and two‐way ANOVA followed by Tukey's multiple comparisons tests for (K,N,O). PAS, periodic acid‐Schiff; qRT‐PCR, quantitative real‐time PCR; ****P ≤ 0.0001. ANOVA, analysis of variance.

To assess the functional capacity of the splenic regenerated thyroid glands, we examined the expression of genes and molecules necessary for thyroid function. Thyroglobulin (Tg) is essential for the synthesis and iodination of thyroid hormones T_3_ and T_4_, and PAS staining (**Figure** **4**A; Figure S5A, Supporting Information) and immunofluorescence staining (Figure 4B; Figure S5B, Supporting Information) revealed the presence of Tg in follicles of tissue sections.^[^ ^29^ ^]^ The mRNA levels were verified by qRT‐PCR^[^ ^30^ ^]^ (Figure 4C), confirming the expression of Tg in the regenerated thyroid gland. Immunofluorescence staining and mRNA expression levels of the specific transcription factors NK2 homology cassette 1 (*NKX2‐1*, also known as *TTF‐1*) (Figure 4D,E, Figure S5B, Supporting Information) and paired cassette 8 (*PAX8*)^[^ ^31^ ^]^ (Figure 4F,G, Figure S5B, Supporting Information), and Immunofluorescence staining for the colocalization of NKX2‐1 and PAX8 in intrasplenic thyroid tissue (Figure 4H, Figure S5B, Supporting Information), demonstrated their expression in the regenerated tissues, which is a characteristic feature of thyroid tissue. In addition, immunofluorescence staining of key proteins (sodium/iodine cotransporter, NIS) (Figure S5B, Supporting Information) and mRNA levels of key genes (*TPO* and *TSHR*) (Figure 4I), involved in the production and function of thyroid hormones, confirmed the expression of proteins and genes essential for thyroid function. In addition, iodine levels were measured, and it was found that the regenerated thyroid tissues had even higher iodine content than native thyroid tissue (Figure 4J), indicating their ability to uptake and store iodine.

To evaluate the functional capabilities of the regenerated thyroid, we conducted a series of in vitro and in vivo tests. First, in an *ex vivo* Forskolin‐stimulated thyroid hormone release test, the isolated thyroid tissues from the spleen (after 16 weeks of growth) demonstrated a response to Forskolin stimulation by releasing the thyroid hormone T_4_, similar to native thyroid tissue (Figure 4K). Second, in a total thyroidectomy mouse model, hypothyroid mice (TH mice) that underwent intra‐splenic thyroid transplantation fully restored their serum hormone levels within 4 weeks after the treatment and maintained stability thereafter. Upon removal of the thyroid‐containing spleens after 16 weeks of hormone level monitoring, hormone levels immediately decreased in these TH mice, confirming that the hormones in the blood were produced by the thyroid glands in the spleen (Figure 4L,M). And there was no thyroid regeneration observed in the larynx (where the thyroid gland was originally located) 16 weeks after thyroidectomy (Figure S5C, Supporting Information). Lastly, the response of mice with regenerated thyroid glands (ST mice) to thyrotropin‐releasing hormone (TRH) treatment was tested. It was found that the mice with regenerated thyroid glands responded well to TRH stimulation, as observed by changes in serum T_4_ levels and body temperature, similar to healthy animals^[^ ^32^ ^]^ (Figure 4N,O).

In conclusion, our findings provide evidence that regenerating the thyroid within the spleen enables the restoration of thyroid functions. The regenerated glands exhibited excellent capacity to maintain normal blood hormone levels and respond to physiological regulatory signals, such as TRH stimulation. This is particularly significant because standard clinical treatment for hypothyroidism with oral L‐T_4_ supplementation lacks the ability to respond to physiological regulation, which is essential for patients.

### 2.5 Restoration of Physiological Homeostasis in Hypothyroid Mice by the Intrasplenic Regenerated Thyroid

In this section, we aimed to assess the ability of splenic regenerated thyroid tissue to restore the functions of the main affected organs in TH mice and compare it with the effects of oral L‐T_4_ treatment. It is known that hypothyroidism affects various physiological systems, and oral L‐T_4_ replacement therapy continues to provide low serum T_3_ levels even when serum T_4_ levels are normal, and is unable to fully reverse all clinical manifestations due to its inability to respond to immediate physiological signals and provide normal regulatory feedback.^[^ ^33^ ^]^

We treated hypothyroid mice with oral L‐T_4_ and transplantation to examine the effects of both treatments. Serum T_4_ levels were corrected but T_3_ levels were less increased in the animals treated with oral medication compared to transplantation. Serum hormone T_3_/T_4_ ratios were normalized in transplanted mice compared to oral drug treatment, which is necessary for hormone action in all organs^[^ ^34^ ^]^ (Figure S6A, Supporting Information). To evaluate the overall physiological state of ST mice (16 weeks post‐transplantation), we performed mRNA sequencing and gene expression profiling in three major organs: brain, heart, and liver. PCA (Figure S6B, Supporting Information), Spearman analysis (Figure S6C, Supporting Information) and heat map (Figure S6D, Supporting Information) suggested that compared to the group receiving oral L‐T_4_ treatment (L‐T_4_ mice), the organs, particularly the hearts and livers of ST mice, exhibited a functioning state closer to that of normal animals (N mice). We examined the expression of important T_3_‐regulated genes (*THRSP1*,^[^ ^35^ ^]^ *Dio1*,^[^ ^36^ ^]^ *ME1*,^[^ ^37^ ^]^ and *Myh6* ^[^ ^38^ ^]^), as well as genes associated with T_3_ malfunctions, (*Per3* ^[^ ^39^ ^]^ and *Ciart* ^[^ ^40^ ^]^ related to circadian rhythm, *Trib3* ^[^ ^41^ ^]^ related to glucose regulation, *Gclc* ^[^ ^42^ ^]^ related to glutathione synthesis, *Cyp2c69* and *Cyp21a1* related to lipid metabolism,^[^ ^43^ ^]^ *Esm1* and *Aplnr* ^[^ ^44^ ^]^ related to angiogenesis) using qRT‐PCR (Figure S7A, Supporting Information).

**Figure 5:**
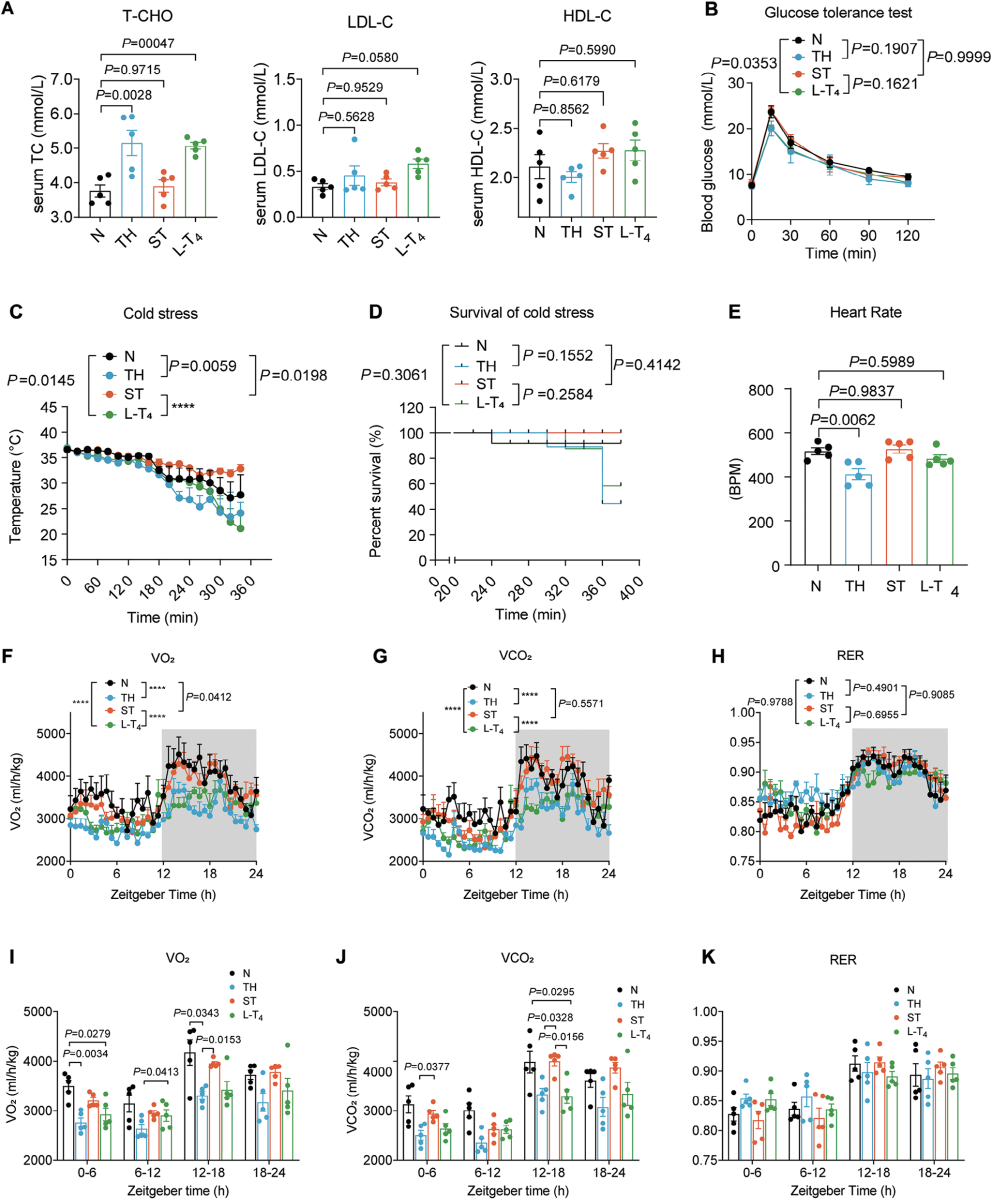
Restoration of physiological homeostasis in hypothyroid mice by the intrasplenic regenerated thyroid. The mice were divided into four groups: N mice (normal healthy mice), ST mice (hypothyroid mice receiving intra‐splenic thyroid regeneration treatment), TH mice (mice with total thyroidectomy), and L‐T_4_ mice (hypothyroid mice receiving oral L‐T_4_ treatment). All measurements were performed 16 weeks after intrasplenic thyroid transplantation in ST mice. A) Serum levels of total cholesterol (T‐CHO), LDL cholesterol (LDL‐C), and HDL cholesterol (HDL‐C) in the mice. *n* = 5 biological replicates. B) Glucose tolerance test in the mice. *n* = 5 biological replicates. C) Body temperature profile and D) survival rate of the mice in the cold stress test. *n* = 5 biological replicates. E) Heart rate of the mice. *n* = 5 biological replicates. F–K) Metabolic cage tests measuring and statistics of F,I) VO_2_, G,J) VCO_2_ and H,K) RER in the mice. *n* = 5 biological replicates. Data are presented as means ± SEM. Statistical analyses were performed using one‐way ANOVA followed by Tukey's multiple comparisons tests for (A,E), and two‐way ANOVA followed by Tukey's multiple comparisons tests (B,C,F,G,H,I,J,K). Survival curve significance was determined by the log‐rank (Mantel‐Cox) test (D). ****P ≤ 0.0001. ANOVA, analysis of variance.

Furthermore, we assessed the capacity of regenerated thyroids to restore liver functions in lipid and glucose metabolism, as dyslipidemia and glucose abnormalities are common manifestations in thyroidectomized patients receiving oral L‐T_4_ treatment.^[^ ^45^ ^]^ We observed that regenerated thyroids successfully restored serum lipid and cholesterol levels (T‐CHO, LDL‐C, and HDL‐C) (**Figure** **5**A) in TH mice to normal levels, whereas L‐T_4_ therapy did not.^[^ ^46^ ^]^ However there was no significant change in glucose tolerance levels (Figure 5B).

Additionally, we assessed the thermoregulation capability of these animals. Hypothyroid mice have impaired body temperature regulation when exposed to cold stress.^[^ ^47^ ^]^ In the cold stress test, ST mice exhibited excellent body temperature maintenance and survival, whereas oral L‐T_4_ treatment had minimal effect and did not rescue the animals from hypothermia‐induced death (Figure 5C,D). Furthermore, the heart rate can be normalized by thyroid regeneration therapy rather than L‐T_4_ replacement (Figure 5E). In addition, the metabolic cage tests also demonstrated that disrupted parameters such as VO_2_ (Figure 5F,I), VCO_2_ (Figure 5G,J), RER (Figure 5H,K), physical activity (Figure S7B,D, Supporting Information), and food intake (Figure S7C,E Supporting Information) in TH mice were effectively corrected by thyroid regeneration treatment rather than L‐T_4_ replacement.^[^ ^48^ ^]^

Furthermore, to exclude the possibility of hyperthyroidism, we performed a special experiment by transplanting the thyroid gland into mice without thyroidectomy (NT mice), effectively providing the animal with “a second thyroid” in the body. The results showed that serum thyroid hormone levels remained normal even under TRH stimulation (Figure S8A, Supporting Information). Heart rate measurements (Figure S8B, Supporting Information) and metabolic cage tests (Figure S8C–F, Supporting Information) did not reveal any symptoms of hyperthyroidism.

Overall, these results demonstrate that intrasplenic regeneration of the thyroid has the remarkable ability to systematically restore physiological functions affected by hypothyroidism. It proves to be superior to the conventional clinical treatment of oral L‐T_4_ replacement therapy in all aspects.

## 3 Discussion

In recent years, there has been a global increase in the incidence of thyroid cancer.^[^ ^49^ ^]^ The standard treatment for thyroid cancer is typically total thyroidectomy. As per current clinical protocols, patients who undergo total thyroidectomy are required to undergo lifelong oral thyroid hormone replacement therapy.^[^ ^2^ ^]^ However, this treatment approach only maintains the basic physiological regulation of thyroid hormones, disregarding the significant number of patients who have a poor response to oral thyroid hormone.^[^ ^50^ ^]^ Moreover, oral thyroid hormone replacement therapy fails to respond to the precise and dynamic nature of thyroid function, resulting in suboptimal health for thyroidectomized patients to some extent.^[^ ^45a^ ^]^ What is more concerning is that this suboptimal state may persist throughout their lives. Theoretically, the restoration of the physiological regulatory function of the thyroid can only be achieved through thyroid regeneration.

Many thyroidectomies are performed in the early stages of thyroid cancer, where only a small portion of the excised thyroid gland contains cancerous lesions. In theory, through comprehensive in vitro screening, the majority of healthy thyroid tissue can be reimplanted into the patient's body to reconstruct thyroid function. Since in situ reconstruction is not feasible, the conventional clinical approach has been to transplant thyroid tissue into muscle. However, this method has limitations in terms of function reconstruction and long‐term maintenance.^[^ ^16c^ ^]^ The transplantation of muscle tissue is primarily chosen for its ease of operation, but it is not an ideal site for transplantation and regeneration.^[^ ^51^ ^]^ The stress experienced by muscle tissue can disrupt the structure of the thyroid, potentially leading to its loss. The key limiting factor for regenerating complex tissues, especially thyroid tissue, is the reconstruction of the vascular system. The thyroid not only requires a basic blood supply for survival but also a complex vascular network to fully respond to physiological signals.^[^ ^17^, ^18^, ^19^ ^]^

The primary reason for selecting the spleen as the site for thyroid regeneration is its abundant blood supply and its loose tissue structure, which can accommodate relatively intact thyroid tissue fragments. Unlike muscle, transplanting thyroid tissue into the spleen does not cause subsequent damage to the transplanted tissue. Our recent study demonstrated the spleen's remarkable regenerative capabilities as an in vivo bioreactor by successfully regenerating liver tissue within it.^[^ ^21^ ^]^ We observed that the transplanted thyroid tissue blocks in the spleen completed vascular system reconstruction within 48 hours, which is a critical factor for the success of our research project. However, we did not anticipate the exceptional regenerative capacity of the transplanted thyroid tissue in the spleen. The adult thyroid gland has limited regenerative ability, and even in cases of partial thyroidectomy, the remaining thyroid tissue does not regenerate to compensate for functional loss. To date, there have been no reports of complete regeneration of thyroid tissue transplanted into muscle or other sites. However, in our study, the thyroid tissue transplanted into the spleen exhibited regrowth and development, reaching a size comparable to that of a natural thyroid gland. This finding holds immense significance for thyroid regeneration because, in clinical practice, only a small portion of the patient's thyroid tissue removed during thyroidectomy may be suitable for transplantation back into their body. Moreover, factors such as cryopreservation and the transplantation process itself may cause a significant amount of tissue death. If the remaining thyroid tissue fails to regenerate and grow, the restoration of thyroid regulatory capacity may not be fully achieved.

Based on our discoveries in the spleen, where transplanted thyroid tissue can fully regenerate and grow, it is theoretically possible to transplant only a small portion of thyroid tissue into the spleen and wait for the appropriate time to completely restore thyroid function. Our data also demonstrate that the newly regenerated thyroid within the spleen not only possesses intact structural characteristics specific to the thyroid but also exhibits the capacity to respond to stimuli by releasing thyroid hormones and maintaining physiological concentrations in the long term. Additionally, it can fully restore various thyroid hormone‐regulated physiological functions, eliminating the various adverse reactions associated with oral thyroid hormone therapy. Undoubtedly, this technological approach will significantly increase its clinical value and feasibility.

The mechanism behind the activation of regenerative capacity in thyroid tissue within the spleen remains unclear.^[^ ^52^ ^]^ Although we propose that the higher content of IGF‐1 within spleen tissue may play a role, this explanation is not comprehensive. Considering the abundant population of immune cells, such as macrophages, within the spleen, the activation of thyroid regeneration may also result from direct interactions between cells. Further systematic research is necessary to elucidate this mechanism fully.

In our previous study on liver regeneration within the spleen, we proposed that the spleen is a dispensable organ,^[^ ^21a^ ^]^ as long‐term clinical studies have shown that splenectomy does not lead to significant health hazards in patients.^[^ ^53^ ^]^ Furthermore, considering the vital physiological importance of the liver, we suggested sacrificing spleen function during liver regeneration. However, this proposition does not apply to thyroid regeneration. On one hand, the physiological significance of the thyroid is much lower compared to that of the liver. On the other hand, our experimental results indicate that regenerating the thyroid within the spleen does not result in the loss of spleen function. This is mainly because the thyroid occupies only a small fraction of the spleen's volume (approximately one‐twentieth). The regenerated thyroid occupies a limited amount of tissue space within the spleen, while the majority of the spleen's tissue structure remains unaffected. This aspect is advantageous for the clinical implementation of this strategy as it eliminates the need to sacrifice spleen function to support thyroid regeneration.

There might be concerns regarding the safety of intra‐splenic injection, taking into account the potential risks of bleeding and the procedural complexities. To address these concerns, we first draw from our extensive experience, having performed hundreds of intrasplenic injections in mouse models.^[^ ^21b^ ^]^ Our observations indicate that the risk of bleeding is minimal, as we did not encounter any bleeding incidents when using standard syringe needles for the injections. Furthermore, it is important to note that the need for spleen translocation surgery is unnecessary in larger animals and humans. Percutaneous intra‐splenic injections guided by type‐B ultrasound are relatively straightforward within the realm of minimally invasive surgery. Nevertheless, these considerations should undergo thorough evaluation in experiments involving larger animals and in clinical studies.

We also conducted simulations of allogeneic transplantation by transferring thyroid tissue between mice with different genetic backgrounds. However, this attempt proved unsuccessful due to natural immune rejection, even in the absence of immunosuppressive drugs. This raises questions about the feasibility of allogeneic transplantation, especially when autologous transplantation is deemed unsafe due to the potential for tumor recurrence. Recent advances in culturing the thyroid in vivo from embryonic stem cells (ESC) and induced pluripotent stem cells (iPSC) offer a promising solution to this challenge.^[^ ^54^ ^]^ ESCs and iPSCs have been induced to form functional thyroid organoids in vivo, displaying thyroid function in mice.^[^ ^30^, ^31^, ^55^ ^]^ Theoretically, by harnessing this technology, patient‐derived iPSCs and organoids may induce minimal immune rejection. However, the road ahead would be long in the clinical application of ESCs or iPSCs, impeded by such factors as low reprogramming efficiency and the potential for new mutations generated during cell reprogramming and culture. To avoid these issues, a thyroid organoid model using adult‐derived stem cells (ASCs) generates functional thyroid follicles and survives after being transplanted into nude mice.^[^ ^54^, ^55^, ^56^ ^]^ However, these methods face several challenges, such as low engraftment rates, immune rejection, and inadequate blood supply. Furthermore, we can explore strategies for integrating iPSC‐organoid/ASC‐organoid technology with our intra‐splenic regeneration approach to address issues related to rapid in vivo integration and functionalization. This is particularly pertinent in light of vascular challenges similar to those addressed in the transplantation endeavors of our current study.

## 4 Conclusion

Our study presents a novel strategy for thyroid regeneration and demonstrates the potential of the spleen's tissue environment to activate the regenerative potential of the thyroid. Thyroid regeneration within the spleen fully restores the natural structure and function of the thyroid at the tissue and physiological levels, providing a powerful and promising approach for restoring thyroid endocrine regulation in patients undergoing thyroidectomy.

## 5 Experimental Section

### Animals

All mice were randomized according to body weight before interventions. C57BL/6J, ICR, and GFP^+^ (C57BL/6JGpt‐H11^em1Cin(CAG‐EGFP‐polyA)^/Gpt) mice at 5 to 8 weeks of age were obtained from Gempharmatech (Jiangsu, China). The mice were housed in a specific pathogen‐free animal facility with controlled temperature, humidity, and a 12 h light/12 h dark cycle. They were provided with standard rodent food and water ad libitum. All animal work was performed with permission from the Institutional Animal Care and Use Committee (IACUC) of Nanjing University overseeing the operation (IACUC number 2203007‐2), and conformed to the Guide for the Care and Use of Laboratory Animals published by the National Institutes of Health.

### Total Thyroidectomy

The mice were anesthetized by intraperitoneal injection of 1.25% Tribromoethanol (Yihe, M2910). The neck of the mice was shaved, and a 1.5 cm longitudinal skin incision was made along the midline, separating the sternocleidomastoid and sternohyoid muscles. By clamping the two muscles with microscopic clamps, the thyroid cartilage and thyroid gland were fully exposed. The thyroid isthmus was carefully removed, followed by the separation of the two sides of the thyroid from the lower to the upper trachea, taking precautions to avoid injury to the recurrent laryngeal nerve and promptly addressing any bleeding. After suturing the skin around the neck, the mice were placed on an electric blanket until they recovered from anesthesia and then returned to a clean cage.

For the treatment of hypothyroidism in mice, L‐T_4_ (MeilunBio, Dalian, China, MB1466) was administered orally at a dose of 1.0 µg per 25 g body weight per day to the thyroidectomized mice. This dosage was determined based on the equivalent daily production rate for L‐T_4_ treatment in mice.^[^ ^34^, ^57^ ^]^

### Translocation of the Spleen

To minimize potential injury during transplantation and facilitate easy observation, spleen translocation surgery was performed, transferring the spleen from the peritoneal cavity to the subcutaneous area without cutting off the blood supply. The spleen translocation procedure followed previously established protocols. ^[^ ^21b^ ^]^ The detailed process was as follows: 1) The mice were anesthetized by intraperitoneal injection of 1% pentobarbital sodium (50 mg kg^−1^ body weight). 2) The left flank hair was removed, and the skin was cleaned with 70% ethanol. 3) A 2.5 cm incision was made in the skin over the spleen. Subsequently, a 1 cm incision was carefully created on the exposed peritoneum, taking care to avoid blood vessels and other organs. 4) The spleen was gently extracted from the abdominal cavity, and the connective tissue between the two blood vessels was removed. The peritoneum was sutured. 5) The skin incision was sutured, and the mice were placed on an electric blanket until they recovered from anesthesia before being returned to a clean cage.

### Tissue Grafting

The thyroid gland of donor mice was removed, and each lobule was divided into three equal parts under a body microscope and trimmed into blocks of approximately 0.2, 0.5, and 0.7 mm in diameter, respectively. And according to the size, the blocks were divided into different groups. Then 1.5 mg tissue fragments were then dispersed in a 75–100 µL solution of 0.75% hyaluronic acid (TargetMol, Boston, Massachusetts, T3762). For subsequent grafts, the 0.2, 0.5, and 0.7 mm tissue blocks were injected using syringes sized 26G, 23G, and 20G, respectively.

One week after spleen translocation, the mouse was anesthetized. The tissue suspension was injected into the spleen using a syringe needle. Alternatively, other transplantation sites such as the lateral thigh muscles or the dorsal subcutis of mice were used. For these sites, a small incision was made, the tissue was cut into smaller fragments and buried, and the incision was then sutured.

For cryopreservation, the thyroid gland was stored in CELLSAVINGTM Cell Freezing Solution (NCM Biotech, Suzhou, China, C40100) at −80 °C for 24 h. Subsequently, the tissue was transferred to liquid nitrogen for seven days before being rapidly thawed for transplantation.

### H&E, PAS, and Masson's Trichrome Staining

The harvested tissues were fixed in 4% paraformaldehyde for 2 hours, followed by dehydration in a gradient alcohol series. Subsequently, the tissues were embedded in paraffin. Sections with a thickness of 5 µm were prepared from the paraffin blocks and subjected to staining using H&E (leagene, Beijing, China, DH0001, DH0055), PAS (Jiancheng, Nanjing, China, D004‐1), and Masson's trichrome staining (KeyGen Biotech, Nanjing, China, KGMST‐8004), following the instructions provided by the respective manufacturers.

The stained sections were observed and photographed using a microscope (NIKON DS‐RI2) at various magnifications.

### Statistics of Epithelial Cells and Thyroid Follicles

For counting the number of thyroid follicular epithelial cells, GFP‐labeled intra‐splenic thyroid tissue was separated using laser techniques. Using a hand‐held laser light and filters (Luyor‐3415RG), the location of GFP‐labeled thyroid tissue in the spleen was observed, and the fluorescent tissue was carefully separated with ophthalmic microforceps. The tissue was then digested into single cells using a combination of collagenase I (Sango Biotech, Shanghai, China, 1 mg mL^−1^, A004194‐0001), collagenase II (Sango Biotech, 1 mg mL^−1^, A004174‐0005), collagenase IV (Sango Biotech, 1 mg mL^−1^, A004186‐0005), and trypsin (Sango Biotech, 1.25 mg mL^−1^, A620627‐0250). The number of cells was counted and compared to the control group using normal thyroid tissue.

To determine the number of follicles in the spleen, the spleens were fixed and embedded in paraffin. Hundreds of serial sections were collected. The follicles in sections were counted at 50–75 µm intervals, and the cumulative count was used to obtain the final number of follicles in the spleen. The volume of follicles or colloids was calculated according to the equation below:^[^ ^25^ ^]^

(1) $$V=BP^{3/2}$$

where *P* represents the sectional area. A *B* coefficient of 1.85 was applied for follicles and 1.382 for colloids, as suggested by Zbigniew Kmieć (1998). The thyroid activation index was calculated by determining the ratio of epithelial volume to colloid volume. Epithelial cell height, follicular sectional areas, and colloidal sectional areas were measured using microscopy (NIKON DS‐RI2).

### Immunohistochemistry

For immunohistochemistry, the sections were deparaffinized and rehydrated. To block endogenous peroxidase activity, the sections were incubated in a 3% hydrogen peroxide solution for 10 minutes. Antigen retrieval was performed by heating the sections in Tris‐EDTA buffer (pH = 9.0) (Solarbio, Beijing, China, C1038) at 95 °C for 20 min, followed by cooling to room temperature for 30 min. Nonspecific antibody binding was blocked by incubating the sections in a 5% BSA (Beyotime Biotechnology, Shanghai, China, ST023) solution for 1 h.

Next, the sections were incubated with the primary antibodies overnight at 4 °C, followed by incubation with the secondary antibody for 1 h. The sections were then stained using a DAB chromogenic substrate kit (brown) (Boster, Wuhan, China, AR1022) for 5 min, and the nuclei were counterstained with hematoxylin (leagene, DH0001) for 40 s. Photomicrographs of the stained sections were captured using a microscope at various magnifications (NIKON DS‐RI2). The primary antibodies used were: rabbit anti‐CaSR (Abcam ab259846; 1:50) and rabbit anti‐PTH (Abcam ab213557; 1:50).

### Flow Cytometry

Spleen samples were analyzed using flow cytometry. The tissue was digested with collagenase I (Sango Biotech, 1 mg mL^−1^) to generate a cell suspension. After erythrocyte lysis, cells were blocked with 5% bovine serum albumin and incubated with fluorescence‐coupled antibodies for 30 min at 4 °C in the dark. Flow cytometry data were acquired using FACS Calibur (BD Biosciences, New Jersey, USA) and analyzed using FlowJo 10.8.1 software (Tree Star, San Diego, USA). Each sample was measured three times according to gating strategy. The following antibodies were used for flow cytometry, following the manufacturer's instructions: FITC anti‐mouse CD45 Antibody (Biolegend, San Diego, CA, USA, 103108, 1:100), Brilliant Violet 711 anti‐mouse CD3 Antibody (Biolegend, 100241, 1:80), Brilliant Violet 421 anti‐mouse CD4 Antibody (Biolegend, 100438, 1:160), APC anti‐mouse CD8a Antibody (Biolegend, 100712, 1:80), PE anti‐mouse CD19 Antibody (Biolegend, 115508,1:80), PE anti‐mouse CD49b (pan‐NK cells) Antibody (Biolegend, 108907, 1:80), PE/Cyanine7 anti‐mouse/human CD11b Antibody (Biolegend, 101215, 1:80), Brilliant Violet 711 anti‐mouse F4/80 Antibody (Biolegend, 123147, 1:40), PE anti‐mouse CD86 Antibody (Biolegend, 105007, 1:20), Alexa Fluor 647 anti‐mouse CD206 (MMR) Antibody (Biolegend, 141712, 1:40), PE anti‐mouse CD31 Antibody (Biolegend, 102407, 1:20), and Alexa Fluor 647 Rabbit monoclonal [EPR3776] to Vimentin (Abcam, ab194719).

### ImmuCC Estimates Relative Immune Cell Composition

Immune CC is an inverse convolution model that utilizes a hypothetical linear relationship between the mixed expression profile and the expression profile of isolated cell types in tissue samples. It is employed to estimate the relative proportions of immune cell types.^[^ ^22^ ^]^ To perform cell composition estimation using Immune CC, the sequencing data were uploaded to the website: (http://bioinfo.life.hust.edu.cn/ImmuCellAI‐mouse/#!/).

### Blood Routine Examination

Blood samples were collected and analyzed using an Auto Hematology Analyzer (BC‐2800 Vet) to determine the counts and measurements of various hematological parameters, including lymphocytes, monocytes, neutrophils, erythrocytes, hemoglobin, hematocrit, mean corpuscular volume, mean corpuscular hemoglobin, mean corpuscular hemoglobin concentration, red cell distribution width, platelets, mean platelet volume, platelet distribution widths, and plateletcrit.

### Immunofluorescence

The tissue slices were placed on adherent slides and subjected to gradient hydration. To block nonspecific binding, a 5% bovine serum albumin (BSA) (Beyotime, ST023) solution was applied at room temperature for 1 h. Heat‐mediated antigen retrieval was performed using Tris‐EDTA buffer (pH = 9.0) (Solarbio, C1038) or improved citrate antigen retrieval solution (pH = 6.0) (Beyotime, P0083) for 20 min at 95 °C, following the instructions of the respective antibodies. The slices were then incubated with primary antibodies overnight, and the corresponding fluorescent secondary antibodies for 1 h at room temperature. Nuclei were stained with DAPI (Beyotime, C1005). Fluorescence imaging was conducted using the ZEISS LSM 980 confocal microscope.

Similar staining protocols were applied to native thyroid tissue and isolated intra‐splenic thyroid. In brief, tissues were cut into 0.7 mm diameter pieces, fixed in 4% paraformaldehyde (PFA) for 2 h, and subjected to the antibody staining protocol with the addition of 0.3% Triton‐X100 (Beyotime, P0096).

The primary antibodies used were as follows: rabbit anti‐Tg (abcam, ab156008; 1:200), rabbit anti‐NKX2‐1 (abcam, ab227652; 1:50), rabbit anti‐PAX8 (Proteintech, Wuhan, China, 10336‐1‐AP, 1:100), mouse anti‐PAX8 (abcam, ab53490, 1:100), rabbit anti‐NIS (Proteintech, 24324‐1‐AP; 1:50), rabbit anti‐COL I (abcam, ab270993; 1:1000), rabbit anti‐COL IV (abcam ab6586, 1:200), anti‐PCNA (abcam, ab92552; 1:50), anti‐Endomucin (EMCN) (Invitrogen HM1008, 1:250), anti‐CD31 (abcam, ab28364, 1:200), rabbit anti‐Cyclin D1 (abcam, ab134175, 1:100), rabbit anti‐CD3 (abcam, ab16669; 1:200), rabbit anti‐F4/80 (Cell Signaling Technology, Boston, USA, 30325s; 1:200), rat anti‐B220 (eBioscience, California, USA, 14‐0452‐82; 1:100) and chicken anti‐GFP (abcam, ab13970; 1:1000). The secondary antibody used were: Goat anti‐Chicken IgY (H+L) Secondary Antibody, Alexa Fluor 546 (Invitrogen, California, USA, A11040, 1:200); Donkey anti‐Rabbit IgG (H+L) Highly Cross‐Adsorbed Secondary Antibody, Alexa Fluor 488, (Invitrogen, A21206, 1:200); Goat anti‐Mouse IgG (H+L) Cross‐Adsorbed Secondary Antibody, Alexa Fluor 647 (Invitrogen, A21235, 1:200); Donkey anti‐Rat IgG (H+L) Highly Cross‐Adsorbed Secondary Antibody, Alexa Fluor 488 (Invitrogen, A21208, 1:200); Donkey anti‐Mouse IgG (H+L) Highly Cross‐Adsorbed Secondary Antibody, Alexa Fluor 488 (Invitrogen, A21202, 1:200); Donkey anti‐Rabbit IgG (H+L) Highly Cross‐Adsorbed Secondary Antibody, Alexa Fluor 546 (Invitrogen, A10040, 1:200).

Data statistics were performed using Image‐Pro Plus version 6.0.

### EdU Cell Proliferation Assay

Thyroid tissues were cultured in RPMI 1640 medium (WISENT, Nanjing, China, 350‐000‐CL) supplemented with PBS, spleen homogenate or 1 ng mL^−1^ IGF‐1 (MedChemExpress, New Jersey, USA, HY‐P7070) for 6 h. After the culture period, the tissues were subjected to EdU staining to detect proliferating cells. EdU labeling was performed using the BeyoClick EdU Cell Proliferation Kit with Alexa Fluor 647 (Beyotime, C0081S) following the manufacturer's instructions.

### ELISA Detection

Tissues from the mouse spleen, muscle, and subcutis were collected and homogenized to extract the supernatant for cytokine analysis. To measure cytokine levels in the serum, blood samples were collected from the mice's orbital venous plexus. The collected blood samples were allowed to coagulate at room temperature and then centrifuged to obtain serum. ELISA kits were used to measure the levels of IGF‐1 (Multi Sciences, Hangzhou, China, EK9131), TNF‐α (Multi Sciences, EK282), and IFN‐γ (Multi Sciences, EK280) in the tissue supernatant and serum samples. The measurements were performed according to the instructions provided with the ELISA kits. The absorbance of the samples was measured at 450 nm using a microplate spectrometer (Varioskan LUX Multifunctional Enzyme Labeler (Microplate Assay)).

### Western Blot

Thyroid tissues were cultured in RPMI 1640 medium (WISENT, Nanjing, China, 350‐000‐CL) supplemented with PBS, spleen homogenate or 1 ng mL^−1^ IGF‐1 (MedChemExpress, New Jersey, USA, HY‐P7070), and the inhibitor of the IGF‐1 receptor (picropodophyllin, AXL1717) (MEC, HY‐15494) for 6 h. Afterward, the tissues were subjected to Western blot analysis.

For the Western blot, tissue samples were lysed in RIPA buffer (Beyotime, P0013B) containing protease inhibitor PMSF (Beyotime, ST2573) at 4 °C for 30 min. The lysates were centrifuged at 12,000 rpm for 10 min, and the protein extracts were collected.

The protein extracts were separated by SDS‐PAGE electrophoresis and transferred to a PVDF membrane using the Mini‐PROTEAN Tetra Electrophoresis System (Bio‐Rad). The PVDF membrane was then incubated overnight at 4 °C with primary antibodies against Cyclin D1 (Abcam, ab134175, 1:10 000) or GAPDH (Proteintech, HRP‐60004, 1:10 000).

To detect the bound antibodies, a horseradish peroxidase (HRP)‐linked anti‐rabbit secondary antibody (Jackson ImmunoResearch, 111‐035‐003) was used. The HRP signal was visualized using 4200SF (Tanon Shanghai). This allowed for the visualization of Cyclin D1 and the internal control protein GAPDH on the PVDF membrane.

### Inhibition of Thyroid Proliferation by AXL1717

Mice 1 week after thyroid transplantation were injected intraperitoneally with AXL1717 (20 mg kg^−1^ day^−1^), and the PCNA signals were detected by immunofluorescence in the intrasplenic thyroid two weeks later.

### Regeneration Thyroid Angiographic

Fluorescence imaging was employed to observe the regenerated thyroid gland in the spleen. In this study, the thyroid gland from C57BL/6J GFP^+^ mouse was transplanted into the spleen of C57BL/6J mice as a donor. To trace the thyroid blood vasculature in the spleen, DyLight*649 Lycopersicon esculentum lectin (VectorLabs, California, USA, DL‐1178‐1) was injected via the tail vein.

Ten minutes after the lectin injection, the mice were anesthetized, and PBS was instilled through the hepatic portal vein to facilitate blood draining and reduce the influence of autofluorescence from erythrocytes. The fluorescence of the spleen, including GFP bioluminescence, was visualized using a ZEISS LSM 980 confocal microscope. The vascular data were analyzed using the Angiogenesis Analyzer plugin of Image J software. This analysis allowed for the statistical evaluation of vascular parameters in the spleen.

### PCR

Total RNA was extracted from natural thyroid, intra‐splenic thyroid, and spleen tissues using TRIzol reagent (Invitrogen, Thermo Fisher Scientific, 15596026). The quantity and quality of the RNA were assessed using NanoDrop Lite spectrophotometry (Thermo Fisher Scientific).

For reverse transcription PCR (RT‐PCR), 1 µg of RNA was reverse transcribed into cDNA using the HiScript III RT SuperMix for qRT‐PCR (+gDNA wiper) kit (Vazyme, R323‐01). PCR amplification was carried out using the ChamQ SYBR qPCR Master Mix (High ROX Permixed) (Vazyme, Q341).

The qRT‐PCR analysis was performed using the Applied Biosystems StepOnePlus Real‐Time PCR System (Thermo Fisher Scientific). Each sample was tested in triplicate, and the experiment was independently repeated three times. The average Ct value of the reference gene (GAPDH mouse) was subtracted from the average Ct value of the target gene. The normalized values, presented as 2^−ΔΔCt^, were used to analyze the relative expression levels of the target genes. Primer sequences were available in Table S2, Supporting Information.

### Determination of Serum T_3_ and T_4_

Blood samples were collected via retro‐orbital collection and allowed to coagulate at room temperature. The samples were centrifuged at 3,500 ×*g* for 15 min to obtain serum. The obtained serum samples were stored at −20 °C until further analysis for hormonal measurements.

The concentrations of Total T_3_ (nmol L^−1^) and Total T_4_ (nmol L^−1^) were determined using the Electrochemiluminescence (ECL) technology. Specifically, the Cobas e 801 Module by Roche Diagnostics was used for the measurement of these hormone levels.

### Forskolin‐Stimulated Thyroid Hormone Release

Native thyroid tissues and isolated intrasplenic thyroid tissues were cultured in RPMI 1640 medium (WISENT, 350‐000‐CL) supplemented with either PBS or 10 × 10^−6^ m forskolin (Selleck, Texas, USA, S2449) for a duration of 3 h. After the incubation period, the culture medium was collected for the determination of T_4_ levels. To calculate T_4_ release, the difference in T_4_ content before and after incubation was measured.

### Determination of Iodine Content

The spleen sample to be tested was lyophilized and pulverized. The tissue was then lysed in a 5% tetramethylammonium hydroxide (TMAH) (Amethyst, 949085) solution and heated at 85 °C for 2 h. After centrifugation, the supernatant was collected. It was diluted and subjected to testing using an inductively coupled plasma mass spectrometer (ICP‐MS) model Aurora M90.

### TRH Stimulation Test

Mice were injected with Protirelin (AbMole, Houston, USA, M5909) at a dose of 0.5 mg per 25 g of body weight or PBS via the tail vein. Subsequently, mouse serum was collected or body temperature was measured. Body temperature data were obtained by measuring anal temperature using an animal temperature maintenance instrument (KEW BASIS FT3400).

### Determination of Serum LDL‐C, HDL‐C, TC, and TG

The blood samples were collected via retro‐orbital collection method, followed by coagulation at room temperature. Subsequently, the samples were centrifuged at 3500 g for 15 min to separate the serum. The concentrations of total cholesterol (T‐CHO), low‐density lipoprotein cholesterol (LDL‐C), and high‐density lipoprotein cholesterol (HDL‐C) were determined using the total cholesterol assay kit (Jiancheng, A111‐1), low‐density lipoprotein cholesterol assay kit (Jiancheng, A113‐1), and high‐density lipoprotein cholesterol assay kit (Jiancheng, A112‐1), respectively, following the manufacturer's instructions.

### Glucose Tolerance Test

The glucose tolerance test was conducted on N, TH, ST, and L‐T_4_ mice after a 12‐hour fasting period. Glucose was administered to the mice via intraperitoneal injection at a dose of 2 g kg^−1^ body weight. Blood samples were collected from the caudal vein at various time points: before glucose administration (0 min) and at 15, 30, 60, 90, and 120 min after administration. Blood glucose levels were measured using a blood glucose meter (Yuwell 550).

### Heart Rate

The mice were anesthetized and heart rate was measured by PowerLab 15T (ADInstruments, PL15T02). The data were recorded by LabChart 7 for 60‐90 s in a steady state. The heart rate was calculated by counting the time taken for 20 heartbeats.

### Cold Stress Test

The cold stress test was conducted on normal, HT, ST, and L‐T_4_ mice. The mice were exposed to a temperature of 4 °C for a duration of 3 h. Throughout this period, the anal temperature of the mice was measured every 20 min using an animal temperature maintenance instrument (KEW BASIS FT3400).

### Quantification and Statistical Analysis

Statistical analyses were conducted using Prism Software (GraphPad, Prism V.8.0.2). The data were presented as sample means with standard errors of the mean (±SEM), as specified in the text and figure legends. The number of samples used is indicated in each case. Differences between groups were assessed using unpaired two‐tailed Student's t‐test, One‐way analysis of variance (ANOVA), or two‐way ANOVA with Tukey's multiple‐comparison post hoc test, as described in the text or figure legends. For survival analysis, the log‐rank (Mantel‐Cox) test was used. A significance level of *P* < 0.05 was considered statistically significant.

## Conflict of Interest

The authors declare no conflict of interest.

## Author Contributions

L.D. initiated the concept and designed the overall studies. L.D. and J.‐F.Z. supervised the work. L.D. and X.‐J.T. led the experiments, collected the overall data. L.D., C.‐M.W., and X.‐J.T. wrote the paper. Z.‐J.Y., Z.‐J.L., Z.‐Z.W., Z.X., C.‐Y.L., and L.‐T.W. performed some of the experiments. All authors contributed to data analysis and provided feedback on the paper.

## Supporting information

Supporting Information

## Data Availability

The data that support the findings of this study are available from the corresponding author upon reasonable request.

## References

[1] J. Sharifi‐Rad , S. Rajabi , M. Martorell , M. D. López , M. T. Toro , S. Barollo , D. Armanini , P. V. T. Fokou , G. Zagotto , G. Ribaudo , R. Pezzani , Fitoterapia 2020, 146, 104640. 32474055 10.1016/j.fitote.2020.104640

[2] W. M. Wiersinga , Nat. Rev. Endocrinol. 2014, 10, 164. 24419358 10.1038/nrendo.2013.258

[3] a) D. A. Hughes , K. Skiadas , D. Fitzsimmons , P. Anderson , A. Heald , BMJ Open 2021, 11, e051702; 10.1136/bmjopen-2021-051702 PMC8647544 34862288

[4] E. Berta , I. Lengyel , S. Halmi , M. Zrínyi , A. Erdei , M. Harangi , D. Páll , E. V. Nagy , M. Bodor , Front. Endocrinol. 2019, 10, 482. 10.3389/fendo.2019.00482 PMC6652798 31379748

[5] a) C. Floriani , B. Gencer , T.‐H. Collet , N. Rodondi , Eur. Heart J. 2018, 39, 503; 28329380 10.1093/eurheartj/ehx050

[6] a) E. M. Wekking , B. C. Appelhof , E. Fliers , A. H. Schene , J. Huyser , J. G. P. Tijssen , W. M. Wiersinga , Eur. J. Endocrinol. 2005, 153, 747; 16322379 10.1530/eje.1.02025

[7] M. Sakr , Y. El‐kerm , W. Abo‐Elwafa , A. Mahmoud , I. Fathi , Head Neck 2018, 40, 34. 29076198 10.1002/hed.24904

[8] R. Sankar , P. G. Roy , M. S. Saund , T. K. Thusoo , D. Roy , Surg. Today 2003, 33, 571. 12884093 10.1007/s00595-003-2557-8

[9] a) F. Medas , M. Tuveri , G. L. Canu , E. Erdas , P. G. Calò , Updates Surg. 2019, 71, 705; 30937820 10.1007/s13304-019-00647-y

[10] I. Chernozemski , K. Christov , Nature 1967, 215, 70. 6053408 10.1038/215070a0

[11] M. Karaman , A. Tuncel , S. Sheidaei , M. H. Karabulut , A. Tatlipinar , Head Neck 2012, 34, 702. 21739520 10.1002/hed.21811

[12] J. H. Raaf , J. F. Van Pilsum , R. A. Good , Ann. Surg. 1976, 183, 146. 1247312 10.1097/00000658-197602000-00011 PMC1344077

[13] B. Papaziogas , A. Antoniadis , C. Lazaridis , J. Makris , R. Kotakidou , G. Paraskevas , T. Papaziogas , J. Surg. Res. 2002, 103, 223. 11922738 10.1006/jsre.2001.6348

[14] I. Gál , I. Mikó , I. Furka , D. Nagy , Magy. Sebesz. 2005, 58, 93. 16018275

[15] S. Monib , H. Habashy , M. Ibrahim , Indian J. Surg. 2021, 83, 1451.

[16] a) T. K. Thusoo , D. Das , J. Am. Coll. Surg. 2003, 196, 663; 10.1016/S1072-7515(03)00103-0 12691953

[17] V. M. L. Ogundipe , J. T. M. Plukker , T. P. Links , R. P. Coppes , Tissue Eng., Part A 2022, 28, 500. 35262402 10.1089/ten.TEA.2021.0221

[18] H.‐H. G. Song , R. T. Rumma , C. K. Ozaki , E. R. Edelman , C. S. Chen , Cell Stem Cell 2018, 22, 340. 29499152 10.1016/j.stem.2018.02.009 PMC5849079

[19] I. M. Colin , J.‐F. Denef , B. Lengelé , M.‐C. Many , A.‐C. Gérard , Endocr. Rev. 2013, 34, 209. 23349248 10.1210/er.2012-1015 PMC3610675

[20] R. E. Mebius , G. Kraal , Nat. Rev. Immunol. 2005, 5, 606. 16056254 10.1038/nri1669

[21] a) C. Liu , L. Wang , M. Xu , Y. Sun , Z. Xing , J. Zhang , C. Wang , L. Dong , Gut 2022, 71, 2325; 34996824 10.1136/gutjnl-2021-325018

[22] a) Z. Chen , L. Quan , A. Huang , Q. Zhao , Y. Yuan , X. Yuan , Q. Shen , J. Shang , Y. Ben , F. X.‐F. Qin , A. Wu , Front. Immunol. 2018, 9, 1286; 29922297 10.3389/fimmu.2018.01286 PMC5996037

[23] J. Rouwkema , A. Khademhosseini , Trends Biotechnol. 2016, 34, 733. 27032730 10.1016/j.tibtech.2016.03.002

[24] A. C. Bianco , G. Anderson , D. Forrest , V. A. Galton , B. Gereben , B. W. Kim , P. A. Kopp , X. H. Liao , M. J. Obregon , R. P. Peeters , S. Refetoff , D. S. Sharlin , W. S. Simonides , R. E. Weiss , G. R. Williams , Thyroid 2014, 24, 88. 24001133 10.1089/thy.2013.0109 PMC3887458

[25] Z. Kmiec , G. Kotlarz , B. Smiechowska , A. Mysliwski , Arch. Gerontol. Geriatr. 1998, 26, 161. 18653134 10.1016/s0167-4943(97)00040-x

[26] a) M. Kalisnik , J. Microsc. 1972, 95, 345; 5067980 10.1111/j.1365-2818.1972.tb03733.x

[27] a) T. Fukushima , T. Nedachi , H. Akizawa , M. Akahori , F. Hakuno , S. Takahashi , Endocrinology 2008, 149, 3729; 18403485 10.1210/en.2007-1443

[28] M. Ren , X. Zhong , C. Y. Ma , Y. Sun , Q. B. Guan , B. Cui , J. Guo , H. Wang , L. Gao , J. J. Zhao , Acta Pharmacol. Sin. 2009, 30, 113. 19060913 10.1038/aps.2008.8

[29] M. Senou , M. J. Costa , C. Massart , M. Thimmesch , C. Khalifa , S. Poncin , M. Boucquey , A. C. Gerard , J. N. Audinot , C. Dessy , J. Ruf , O. Feron , O. Devuyst , Y. Guiot , J. E. Dumont , J. Van Sande , M. C. Many , Am. J. Physiol.: Endocrinol. Metab. 2009, 297, E438. 19435853 10.1152/ajpendo.90784.2008

[30] J. Liang , J. Qian , L. Yang , X. Chen , X. Wang , X. Lin , X. Wang , B. Zhao , Adv. Sci. 2022, 9, e2105568. 10.1002/advs.202105568 PMC8948548 35064652

[31] a) F. Antonica , D. F. Kasprzyk , R. Opitz , M. Iacovino , X. H. Liao , A. M. Dumitrescu , S. Refetoff , K. Peremans , M. Manto , M. Kyba , S. Costagliola , Nature 2012, 491, 66; 23051751 10.1038/nature11525 PMC3687105

[32] M. Yamada , Y. Saga , N. Shibusawa , J. Hirato , M. Murakami , T. Iwasaki , K. Hashimoto , T. Satoh , K. Wakabayashi , M. M. Taketo , M. Mori , Proc. Natl. Acad. Sci. USA 1997, 94, 10862. 9380725 10.1073/pnas.94.20.10862 PMC23510

[33] J. Jonklaas , A. C. Bianco , A. J. Bauer , K. D. Burman , A. R. Cappola , F. S. Celi , D. S. Cooper , B. W. Kim , R. P. Peeters , M. S. Rosenthal , A. M. Sawka , Thyroid 2014, 24, 1670. 25266247 10.1089/thy.2014.0028 PMC4267409

[34] H. F. Escobar‐Morreale , M. J. Obregon , F. Escobar del Rey , G. Morreale de Escobar , J. Clin. Invest. 1995, 96, 2828. 8675653 10.1172/JCI118353 PMC185993

[35] J. Ren , N. Xu , H. Zheng , W. Tian , H. Li , Z. Li , Y. Wang , Y. Tian , X. Kang , X. Liu , Sci. Rep. 2017, 7, 10243. 28860448 10.1038/s41598-017-08452-6 PMC5579026

[36] a) C. Luongo , M. Dentice , D. Salvatore , Nat. Rev. Endocrinol. 2019, 15, 479; 31160732 10.1038/s41574-019-0218-2

[37] X. G. Luong , S. K. Stevens , A. Jekle , T. I. Lin , K. Gupta , D. Misner , S. Chanda , S. Mukherjee , C. Williams , A. Stoycheva , L. M. Blatt , L. N. Beigelman , J. A. Symons , P. Raboisson , D. McGowan , K. Vandyck , J. Deval , PLoS One 2020, 15, e0240338. 33306682 10.1371/journal.pone.0240338 PMC7732128

[38] a) F. Forini , G. Nicolini , C. Kusmic , R. D'Aurizio , A. Mercatanti , G. Iervasi , L. Pitto , Cells 2020, 9, 2155; 32987653 10.3390/cells9102155 PMC7598656

[39] a) L. V. M. de Assis , L. Harder , J. T. Lacerda , R. Parsons , M. Kaehler , I. Cascorbi , I. Nagel , O. Rawashdeh , J. Mittag , H. Oster , eLife 2022, 11, e79405; 35894384 10.7554/eLife.79405 PMC9391036

[40] U. Bagchi , S. T. Gegnaw , N. Milicevic , C. Sandu , J. B. Ten Brink , A. Jongejan , D. Hicks , P. D. Moerland , M. P. Felder‐Schmittbuhl , A. A. Bergen , Biochim. Biophys. Acta, Gene Regul. Mech. 2020, 1863, 194623. 32795630 10.1016/j.bbagrm.2020.194623

[41] S. Prudente , G. Sesti , A. Pandolfi , F. Andreozzi , A. Consoli , V. Trischitta , Endocr. Rev. 2012, 33, 526. 22577090 10.1210/er.2011-1042 PMC3410226

[42] a) J. C. Pedemonte , R. Vargas , V. Castillo , T. Hodali , S. Gutierrez , G. Tapia , I. Castillo , L. A. Videla , V. Fernandez , RSC Adv. 2015, 5, 26209;

[43] C. Liddle , B. J. Goodwin , J. George , M. Tapner , G. C. Farrell , J. Clin. Endocrinol. Metab. 1998, 83, 2411. 9661620 10.1210/jcem.83.7.4877

[44] D. T. Paik , S. Cho , L. Tian , H. Y. Chang , J. C. Wu , Nat. Rev. Cardiol. 2020, 17, 457. 32231331 10.1038/s41569-020-0359-y PMC7528042

[45] a) M. D. Ettleson , A. C. Bianco , J. Clin. Endocrinol. Metab. 2020, 105, e3090; 32614450 10.1210/clinem/dgaa430 PMC7382053

[46] Y. Tasaki , Y. Taguchi , T. Machida , T. Kobayashi , Congenital Anomalies 2010, 50, 186. 20608948 10.1111/j.1741-4520.2010.00287.x

[47] C. J. Gordon , P. Becker , B. Padnos , Am. J. Physiol.: Regul., Integr. Comp. Physiol. 2000, 279, R2066. 11080070 10.1152/ajpregu.2000.279.6.R2066

[48] a) J. E. Silva , Endocrinology 2010, 151, 4;20028877

[49] M. Li , L. Dal Maso , S. Vaccarella , Lancet Diabetes Endocrinol. 2020, 8, 468. 32445733 10.1016/S2213-8587(20)30115-7

[50] B. Biondi , L. Wartofsky , J. Clin. Endocrinol. Metab. 2012, 97, 2256. 22593590 10.1210/jc.2011-3399

[51] M. F. Sakr , in Thyroid Auto‐Transplantation (Ed: M. F. Sakr ), Springer International Publishing, Cham, Switzerland 2020, Ch. 10.

[52] a) J. Lee , S. Yi , J. Y. Chang , Y. E. Kang , H. J. Kim , K. C. Park , K. J. Yang , H. J. Sul , J. O. Kim , H. S. Yi , X. Zhu , S. Y. Cheng , M. Shong , Lab. Invest. 2017, 97, 478; 28112758 10.1038/labinvest.2016.158 PMC7886286

[53] a) N. Vianelli , M. Galli , A. de Vivo , T. Intermesoli , B. Giannini , M. G. Mazzucconi , T. Barbui , S. Tura , M. Baccarani , Haematologica 2005, 90, 72; 15642672

[54] a) V. M. L. Ogundipe , J. T. M. Plukker , T. P. Links , R. P. Coppes , Tissue Eng., Part A 2022, 28, 500; 35262402 10.1089/ten.TEA.2021.0221

[55] a) A. A. Kurmann , M. Serra , F. Hawkins , S. A. Rankin , M. Mori , I. Astapova , S. Ullas , S. Lin , M. Bilodeau , J. Rossant , J. C. Jean , L. Ikonomou , R. R. Deterding , J. M. Shannon , A. M. Zorn , A. N. Hollenberg , D. N. Kotton , Cell Stem Cell 2015, 17, 527; 26593959 10.1016/j.stem.2015.09.004 PMC4666682

[56] J. van der Vaart , L. Bosmans , S. F. Sijbesma , K. Knoops , W. J. van de Wetering , H. G. Otten , H. Begthel , I. H. M. B. Rinkes , J. Korving , E. G. W. M. Lentjes , C. Lopez‐Iglesias , P. J. Peters , H. M. van Santen , M. R. Vriens , H. Clevers , Proc. Natl. Acad. Sci. USA 2021, 118, e2117017118 34916298 10.1073/pnas.2117017118 PMC8713972

[57] H. F. Escobar‐Morreale , F. E. del Rey , M. J. Obregon , G. M. de Escobar , Endocrinology 1996, 137, 2490. 8641203 10.1210/endo.137.6.8641203

