# Supplementary material for: Regeneration of Thyroid Glands in the Spleen Restores Homeostasis in Thyroidectomy Mice
Source: Adv Sci (Weinh). 2023 Dec 7;11(6):2305913. doi: 10.1002/advs.202305913 (PMC10853707; doi:10.1002/advs.202305913)
Supplement: Supplementary file 1 — Supporting Information [file ADVS-11-2305913-s001.pdf]

ADVANCED SCIENCE
Open Access

Supporting Information

for *Adv. Sci.*, DOI 10.1002/advs.202305913

Regeneration of Thyroid Glands in the Spleen Restores Homeostasis in Thyroidectomy Mice

Xue-Jiao Tian, Zhi-Jie Yin, Zhen-Jiang Li, Zhen-Zhen Wang, Zhen Xing, Chun-Yan Liu, Lin-Tao Wang, Chun-Ming Wang, Jun-Feng Zhang and Lei Dong**

Supporting Information

Regeneration of Thyroid Glands in the Spleen Restores Homeostasis in Thyroidectomy Mice

Xue-Jiao Tian, Zhi-Jie Yin, Zhen-Jiang Li, Zhen-Zhen Wang, Zhen Xing, Chun-Yan Liu, Lin-Tao Wang, Chun-Ming Wang, Jun-Feng Zhang, Lei Dong**

X.-Jiao Tian, Z.-Jie Yin, Z.-Jiang Li, Z.-Zhen Wang, Z. Xing, C.-Yan Liu, L.-Tao Wang, J.-Feng Zhang, L. Dong
State Key Laboratory of Pharmaceutical Biotechnology, School of Life Sciences
Nanjing University
Nanjing, Jiangsu 210023, China.
Email: L.D. (leidong@nju.edu.cn); J.Z. (jfzhang@nju.edu.cn)

C.-Ming Wang
State Key Laboratory of Quality Research in Chinese Medicine, Institute of Chinese Medical Sciences, University of Macau,
Taipa, Macau SAR.

L. Dong
National Resource Center For Mutant Mice,
Nanjing, 210023, China.

Z. Xing, L. Dong
Wuxi Xishan NJU Institute of Applied Biotechnology
Anzhen Street, Xishan District, Wuxi 214101, China.

L. Dong
Chemistry and Biomedicine Innovative Center, Nanjing University, Nanjing, Jiangsu 210023, China.

Supplementary Figures

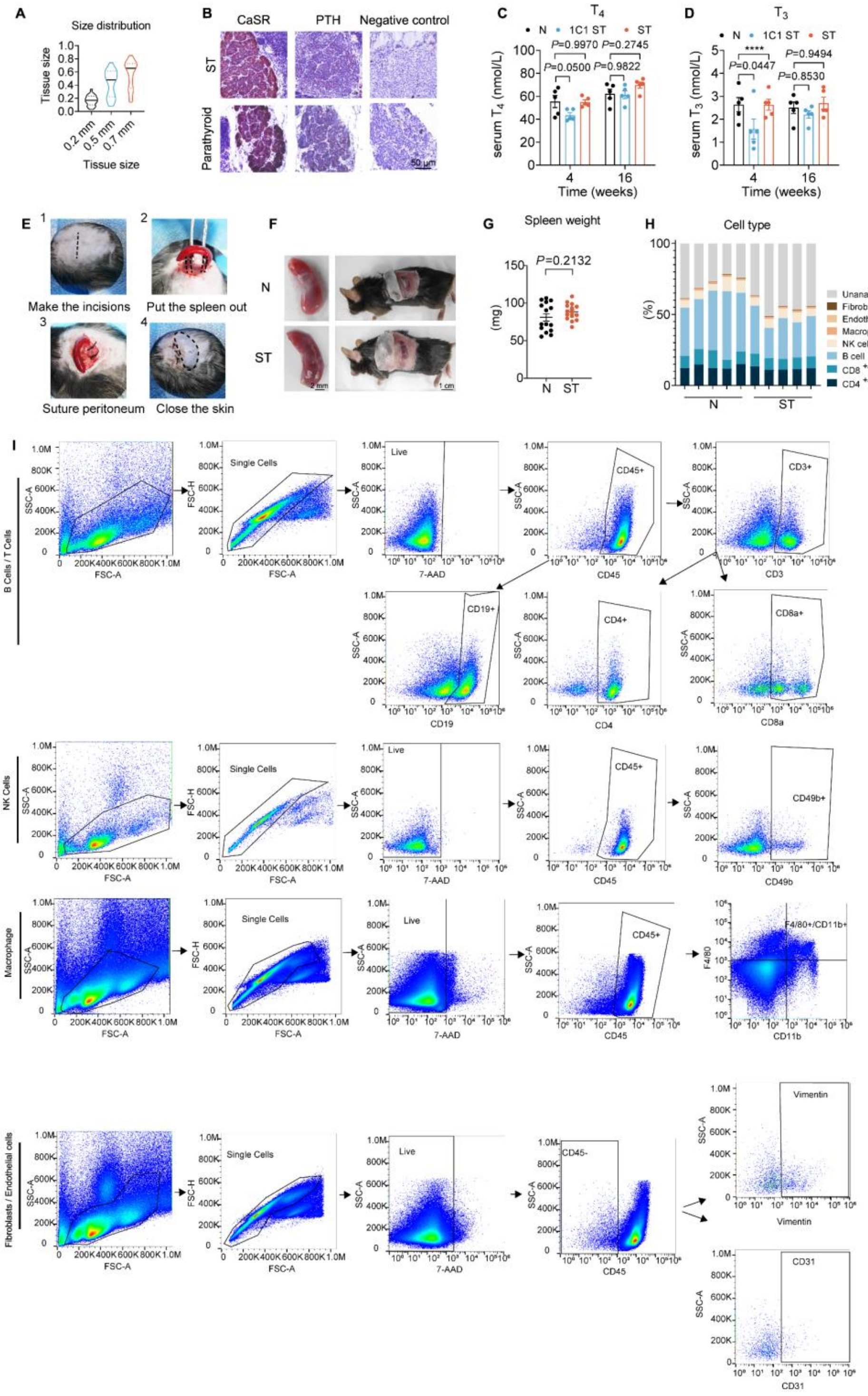

Figure S1. Thyroid Transplantation into Translocated Mouse Spleens. A) Size distribution of chopped thyroid tissue. Thyroid suspensions of 0.2mm, 0.5mm and 0.7mm sizes were prepared, and the size of 20 of these tissues were randomly counted. *n*=20. B) Immunohistochemical staining for parathyroid. Immunohistochemical staining for parathyroid markers PTH and CaSR and negative control. Regenerated parathyroid specimens were harvested from mice 4 weeks after intra-splenic transplantation (ST) of parathyroid glands of the same genotype for immunohistochemical staining. C,D) Serum

hormone recovery in intrasplenic transplantation of frozen-thawed thyroid (1C1ST) and unfrozen thyroid (ST) mice. Serum was collected from mice after thyroidectomy, followed by intra-splenic transplantation of frozen-thawed thyroid and unfrozen thyroid at 4 and 16 weeks for determination of C) T_3 and D) T_4 concentrations by Electrochemiluminescence (ECL) technology, with N mice as the control. *n*=5. E) Major steps in spleen translocation. In Figure 1, an incision is made at the black dashed position; in Figure 2, the vessels are labeled with black dashed lines to mark the vessel position; in Figure 4, the black dashed lines mark the spleen position. F,G) F) Morphology of the spleen after transplantation without (upper) or with (lower) a transplanted thyroid and G) mean spleen weight in ST and normal mice at week 4. *n*=15. Specimens were harvested from the spleens of mice 4 weeks after intra-splenic transplantation of the same genotype of the thyroid (ST), with N mice as the control. H) Proportions of different cell populations in normal or thyroid-transplanted spleens 4 weeks after the transplantation. *n*=5. I) The Gating strategy for flow cytometry. T cell and B cell, NK cell, macrophage, fibroblast, and endothelial cell. Data are presented as means ± SEM. Statistical analyses were performed using two-way ANOVA followed by *Tukey's* multiple comparisons tests (C and D) and *Student's* t-test (G). PTH, parathormone; CaSR, calcium-sensing receptor. ANOVA; analysis of variance, 1C1 ST denotes mice transplanted with frozen-thawed thyroid glands.

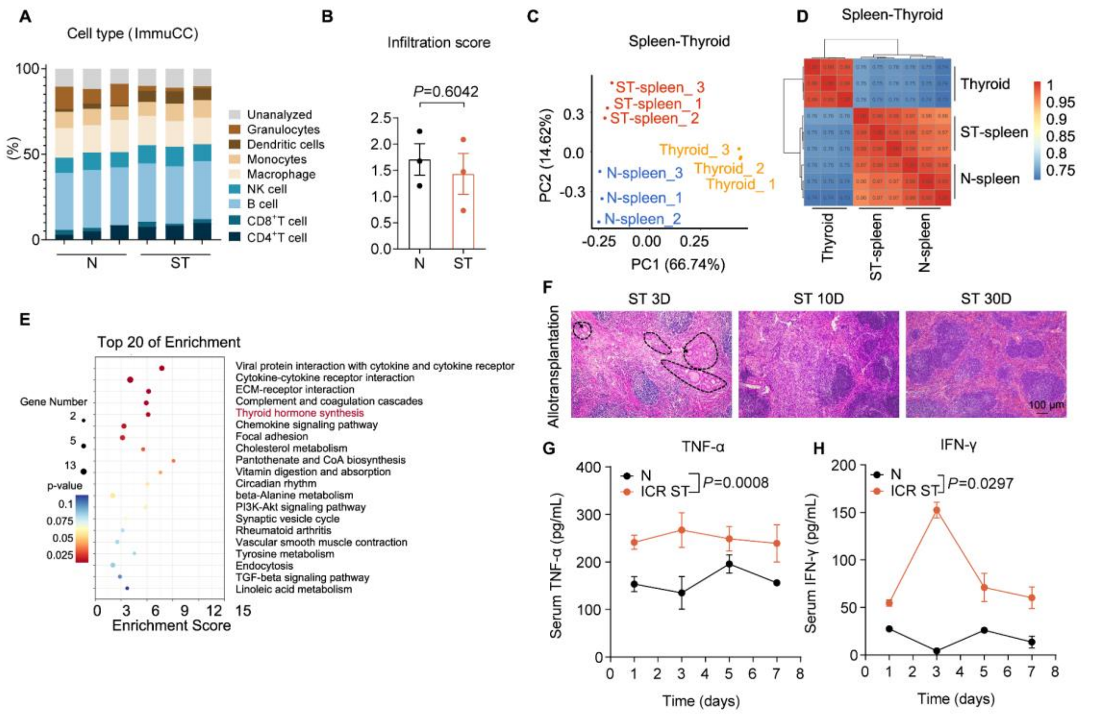

Figure S2. Analysis of Thyroid Autotransplantation and Allotransplantation into Mouse Spleens. Spleen specimens were harvested 4 weeks after intra-splenic transplantation from mice with the same genotype of thyroid (ST), and splenocytes were subjected to flow cytometry to determine cell proportions, with normal spleens (N) as a control. A,B) A) ImmuCC estimates the composition of immune cells from spleen expression profiles, and B) calculates the immune infiltration score. *n*=3. Splenic sequencing data of N and ST were used to predict their immune cell infiltration by uploading to the ImmuCC web server. C) PCA analysis of N-spleen (control), ST-spleen, and thyroid. *n*=3. D) Spearman correlation analysis of gene expression profiles in the N-spleen, ST-spleen, and thyroid. *n*=3. E) KEGG analysis of upregulated enriched genes showing enrichment in the thyroid hormone synthesis pathway. Genome-wide microarray mapping of the spleen (ST-spleen) 4 weeks after thyroid transplantation, with thyroid and normal spleen (N-spleen) as controls. F) H&E staining of thyroid grafts transplanted into the spleens (ICR ST). The black arrows indicate the follicular structure and the black dashed curves outline the ST tissue. Thyroid glands of ICR mice were intra-splenic transplanted in C57 mice, and spleen specimens were harvested at 3, 10, and 30 posts after transplantation and subjected to H&E staining. G,H) Serum cytokine concentrations of G) TNF-α and H) INF-γ in mice transplanted with thyroid glands from donors with different genotypes, with N mice as the control. *n*=3. Thyroid glands of ICR mice were intra-splenic transplanted in C57 mice, and serum samples were collected at 1, 3, 5, and 7 days after transplantation. TNF-α and IFN-γ concentrations were measured by ELISA. Data are presented as means ± SEM. Statistical analyses were performed using *Student's* t-test (B, G, H). ANOVA; analysis of variance, ICR ST denotes mice transplanted with thyroid glands from donors with different genotypes.

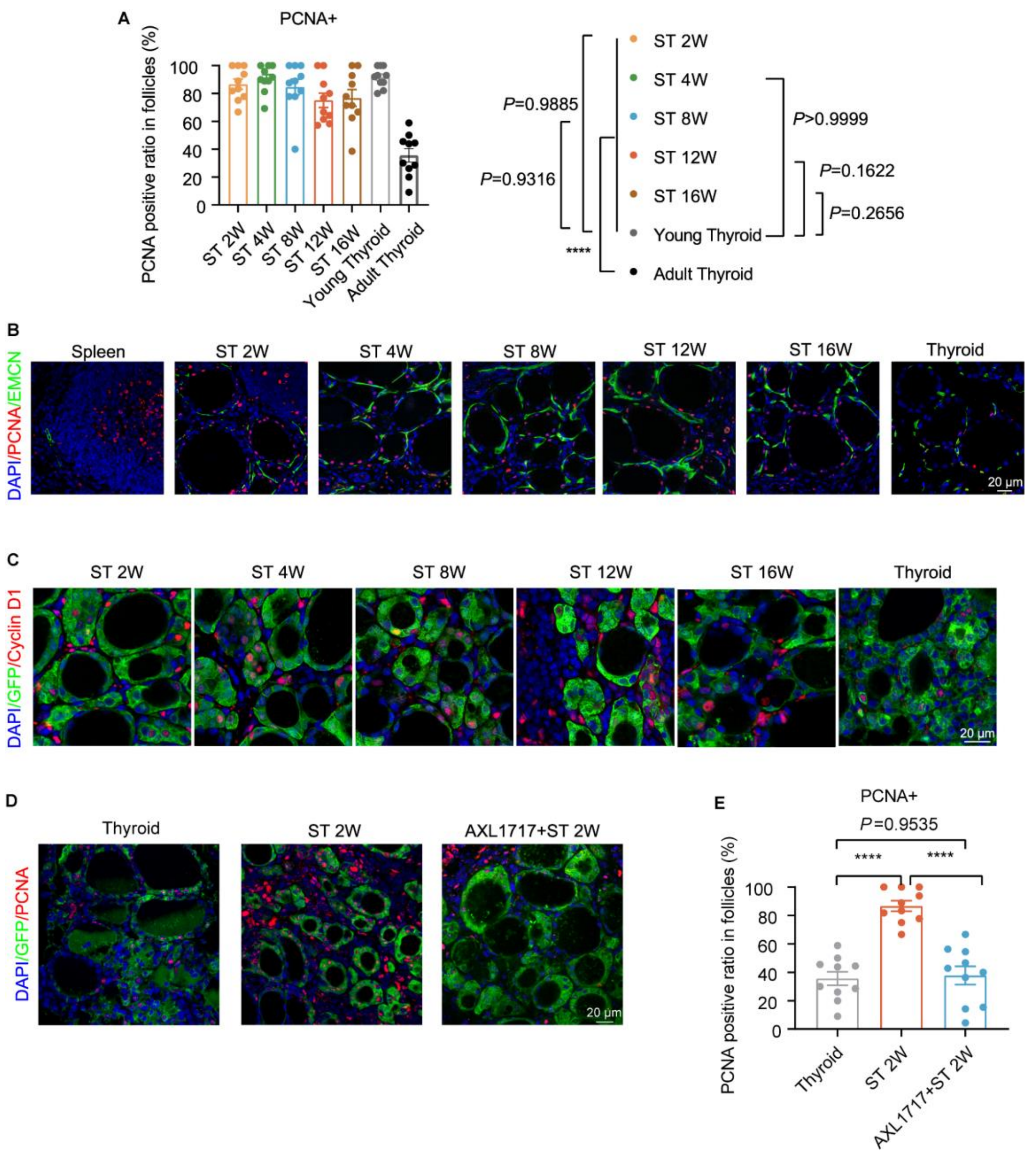

Figure S3. **Regeneration of Transplanted Thyroid Tissue in the Spleen.** A) quantification statistics for the co-localization of PCNA with GFP. Proliferation of thyroid tissue was assessed by calculating the percentage of PCNA-positive nuclei in the nuclei of visible cells in each GFP$^+$ follicle. B) Immunofluorescence staining of EMCN and PCNA expression in the intra-splenic regenerated thyroid tissue at weeks 4 to 16. C) Representative immunofluorescence images for Cyclin D1 in intra-splenic regenerated thyroid tissue (ST). Co-localization of Cyclin D1 and donor-sourced GFP-labeled thyroid was detected by immunofluorescence staining of splenic tissue sections from mice intrasplenic transplanted at 2 to 16 weeks. D,E) The proliferative activity of intra-splenic thyroid after the inhibition of IGF-1 by IGF-1R inhibitor (AXL1717) was evaluated by PCNA D) immunofluorescence staining (co-staining with GFP to identify thyroid tissues from GFP transgenic mice) and E) quantification statistics for the co-localization of PCNA with GFP. Data are presented as

means ± SEM. Statistical analyses were performed using one-way ANOVA followed by *Tukey's* multiple comparisons tests (A and E). ANOVA; analysis of variance.

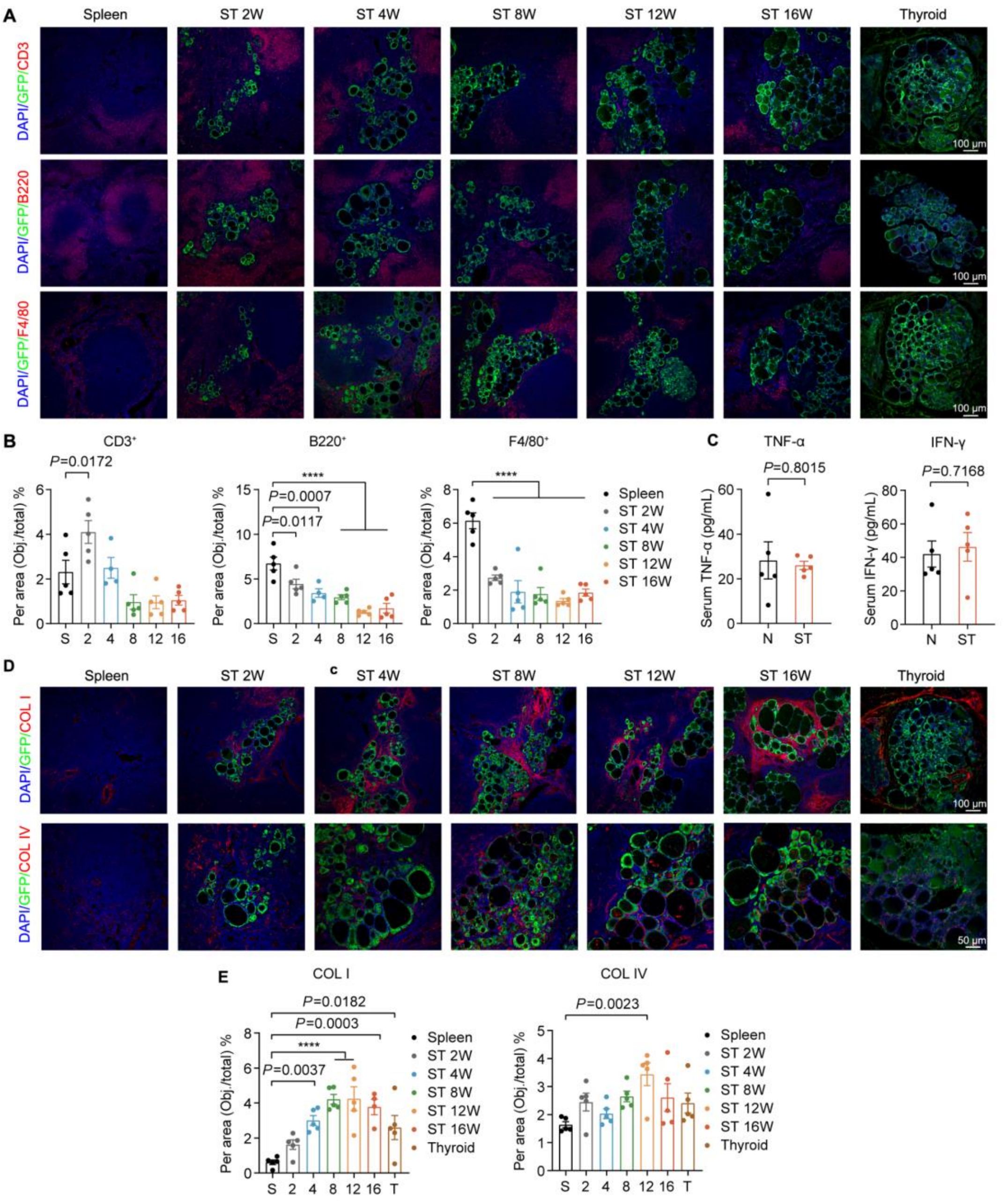

Figure S4. Independence of the thyroid gland in the spleen. A) Representative images of immunofluorescence for T cells ($CD3^+$), B cells ($B220^+$), and macrophages ($F4/80^+$) in the intra-splenic regenerated thyroid tissue (ST) spleens. Expression of immune cell markers in the spleen was determined by immunofluorescence staining of splenic tissue sections from mice with intrasplenic transplantation of the same genotype of thyroid at 2 to 16 weeks. B) Fluorescence statistics of the immunofluorescence staining for T cells, macrophages, and B cells in the intra-splenic regenerated thyroid tissue (ST) spleens. n=5. Data statistics were performed using Image-Pro Plus version 6.0. C) Serum cytokine concentrations of TNF-α and INF-γ in mice transplanted with thyroid glands. n=5. Serum samples were collected from mice intrasplenically transplanted with the same genotype of thyroid at 7 days after transplantation, and TNF-α and IFN-γ concentrations were measured by ELISA. D) Representative images of immunofluorescence for collagen type I (COL I) and collagen type IV (COL IV) in the intra-splenic

regenerated thyroid tissue (ST) spleens. Co-localization of collagen and host-derived GFP-labeled thyroid in the spleen was detected by immunofluorescence staining of splenic tissue sections from mice with intra-splenic regenerated thyroid at 2 to 16 weeks. E) Fluorescence statistics of the immunofluorescence staining for collagen type I and collagen type IV in the intra-splenic regenerated thyroid tissue (ST) spleens at different time points (weeks 2 to 16) after transplantation. n=5. Data statistics were performed using Image-Pro Plus version 6.0. Data are shown as means ± SEM. Statistical analyses were performed using One-way ANOVA followed by *Tukey's* multiple comparisons tests for (B, E); *Student's* t-test for (C). ****$P \leq 0.0001$. ANOVA; analysis of variance.

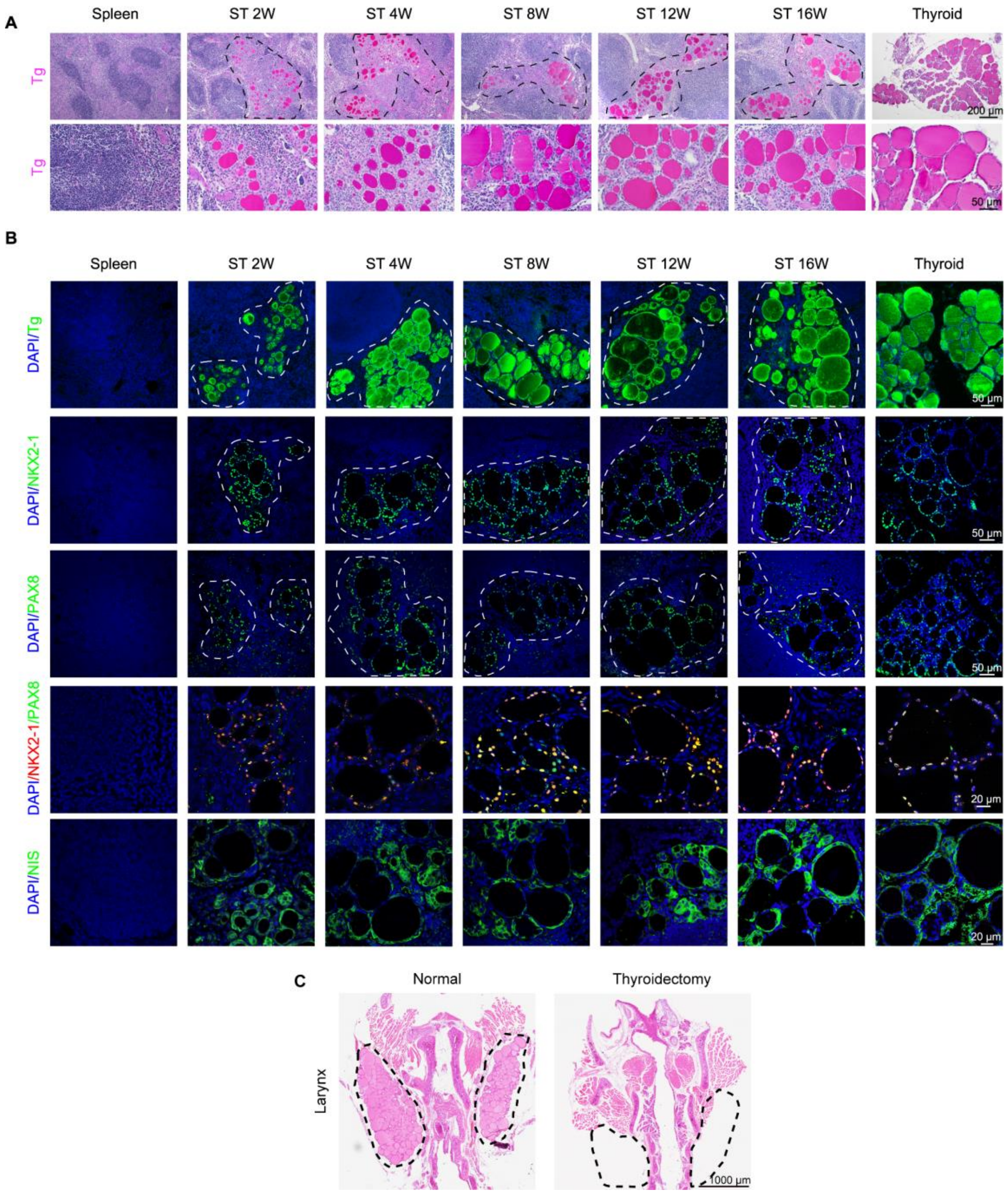

Figure S5. Representative images of PAS and immunofluorescence of thyroid markers. A) PAS staining labels thyroglobulin in the intra-splenic regenerated thyroid tissue (ST), showing the overall (upper) and local (lower) staining. The black dashed curves outline the ST tissue. Spleen specimens were harvested from mice with intra-splenic regenerated thyroid at 2 to 16 weeks. B) Expression of specific thyroid markers Tg, NKX2-1, PAX8, and NIS in the intra-splenic regenerated thyroid tissue (ST) by immunofluorescence staining of splenic tissue sections. The white dashed curves outline the ST tissue. Spleen specimens were harvested from mice with intra-splenic regenerated thyroid at 2 to 16 weeks. C) H&E staining of the larynx (the spot where the thyroid gland used to be) of normal and total thyroidectomy mice.

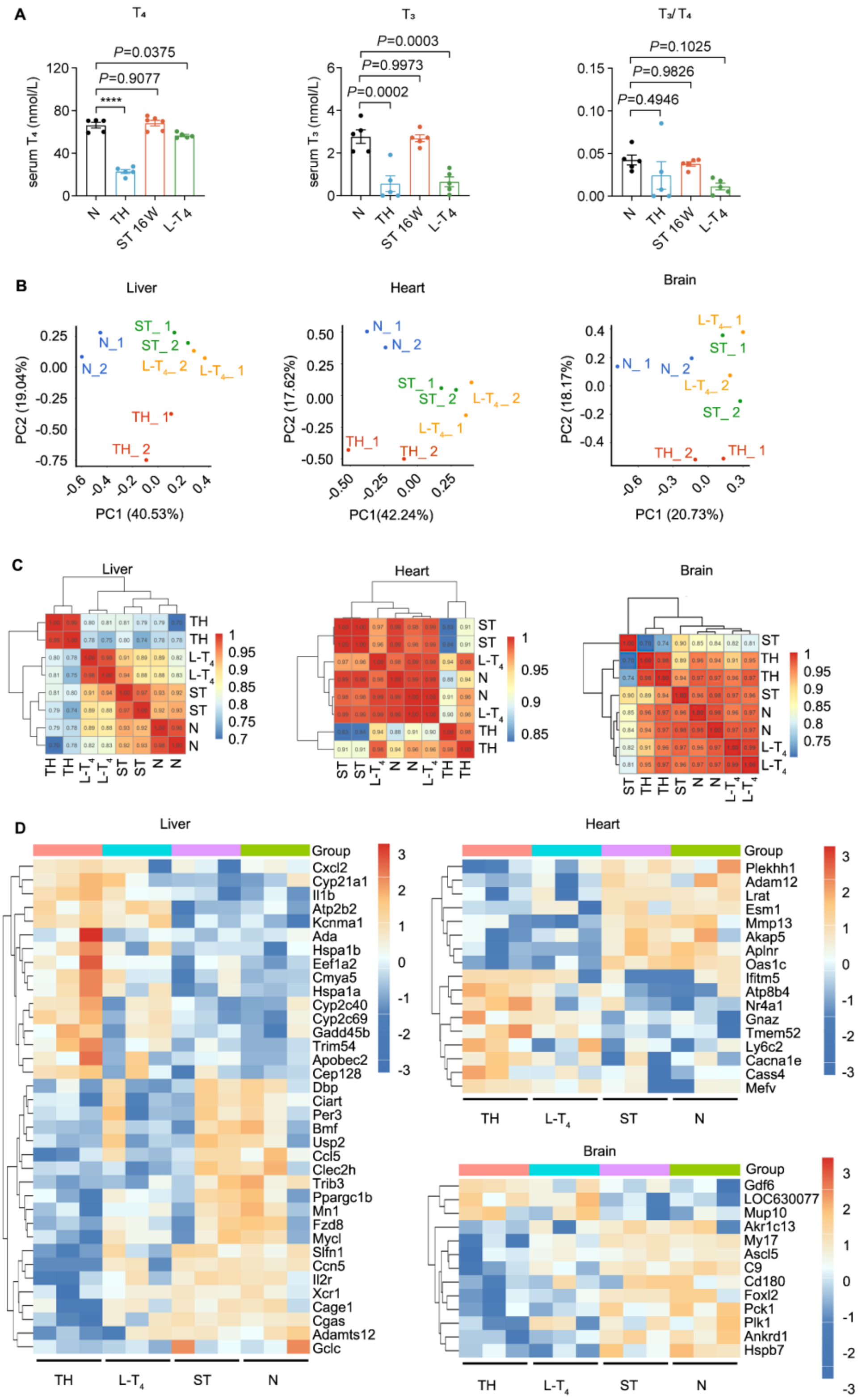

Figure S6. The Effects of the Intra-splenic Thyroids and L-T₄ Treatment on Serum Hormone Level and Functional Gene Expression Profile in Liver, Heart, and Brain in the Hypothyroid Mice. The mice were divided into four groups: N mice (normal healthy mice), ST mice (hypothyroid mice receiving intra-splenic thyroid regeneration treatment), TH mice (mice with total thyroidectomy), and L-T_4 mice (hypothyroid mice receiving oral L-T_4 treatment). All measurements were performed 16 weeks after

intra-splenic thyroid transplantation in ST mice. A) Serum levels of T_4, T_3, and the T_3/T_4 ratio in the mice 16 weeks post-transplantation. *n*=5 biological replicates. B) PCA analysis of gene expression profiles by RNAseq in the brain, heart, and liver of the mice. *n*=2 biological replicates. C) Spearman correlation analysis of the gene expression profile by RNAseq in the brain, heart, and liver of the mice from different groups (N mice, ST mice, TH mice, and L-T_4 mice) at 16 weeks post-intra-splenic thyroid transplantation. *n*=2. Numerical values of correlation coefficients are presented in the corresponding boxes. D) Heat map showing the gene expression levels significantly changed in the heart, brain, and liver of the mice from different groups. *n*=3 biological replicates. Blue to red represent the relative expression levels from low to high. Data are presented as means ± SEM. Statistical analyses were performed using one-way ANOVA followed by *Tukey's* multiple comparisons tests for (A). ****$P \leq 0.0001$; ANOVA; analysis of variance.

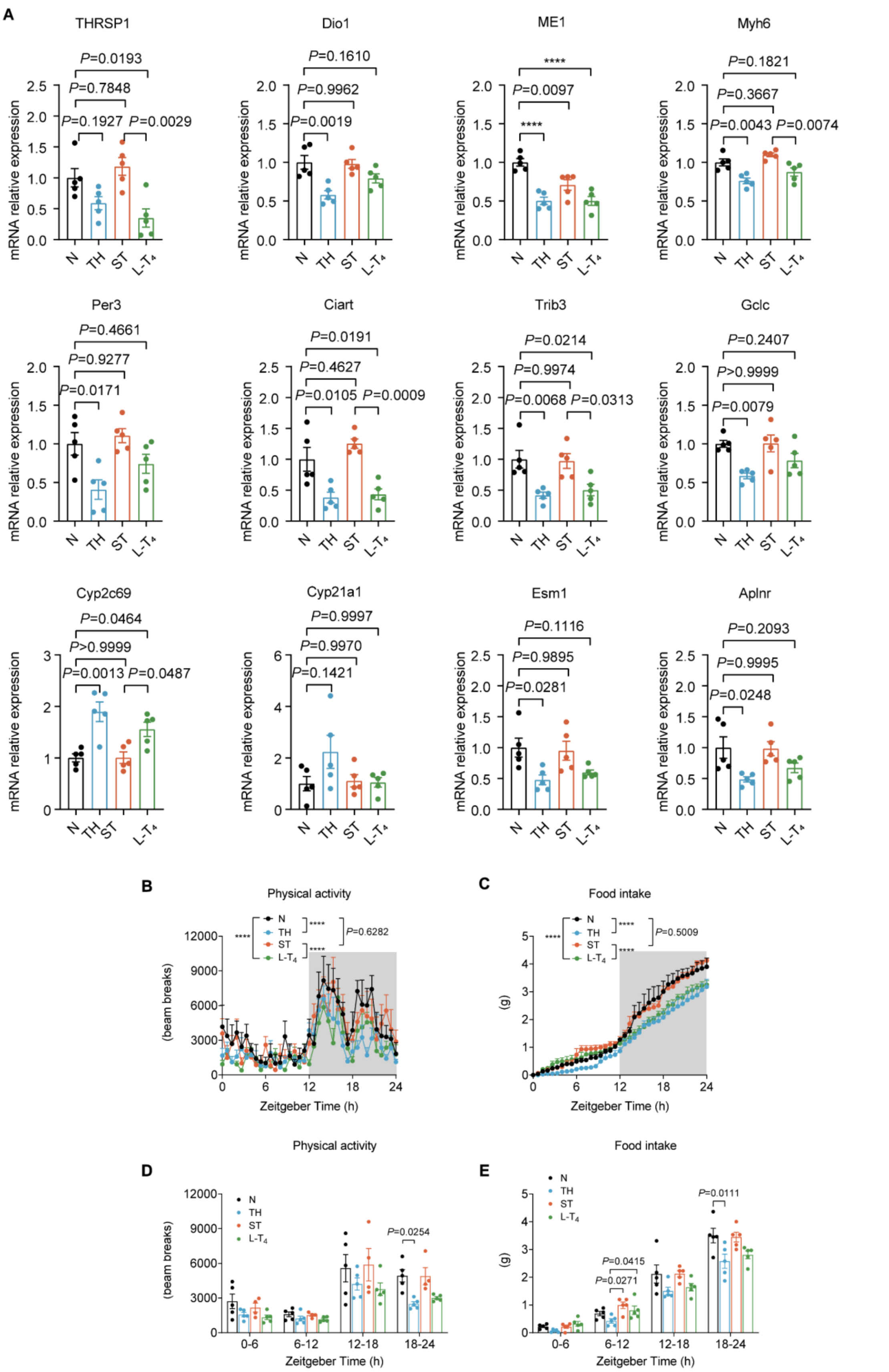

Figure S7. Gene regulation of intra-splenic regenerated thyroid in the liver, heart, and brain. The mice were divided into four groups: N mice (normal healthy mice), ST mice (hypothyroid mice receiving intra-splenic thyroid regeneration treatment), TH mice (mice with total thyroidectomy), and L-T_4 mice

(hypothyroid mice receiving oral L-T_4 treatment). All measurements were performed 16 weeks after intra-splenic thyroid transplantation in ST mice. A) Expression levels of key thyroxine-regulated genes and genes associated with T_3 malfunctions examined by qRT-PCR in the liver (*THRSP1*, *Dio1*, *ME1*, *Per3*, *Cyp2c69*, *Trib3 CXCL2*, *Cyp21a1*, *Cyp2c40*, *Ciart*, and *Gclc*) and heart (*Myh6*, *Esm1, Aplnr*) of the mice from different groups at 16 weeks post-intra-splenic thyroid transplantation. n=5 biological replicates. B-E) Metabolic cage tests measuring and statistics for B,D) physical activity and C,E) food intake in the mice at 16 weeks post-intra-splenic thyroid transplantation.. n=5 biological replicates. Data are presented as means ± SEM. Statistical analyses were performed using one-way ANOVA followed by *Tukey's* multiple comparisons tests (A); two-way ANOVA followed by *Tukey's* multiple comparisons tests (B, C, D and E). ****$P \leq 0.0001$. qRT-PCR; quantitative real-time PCR; ANOVA; analysis of variance.

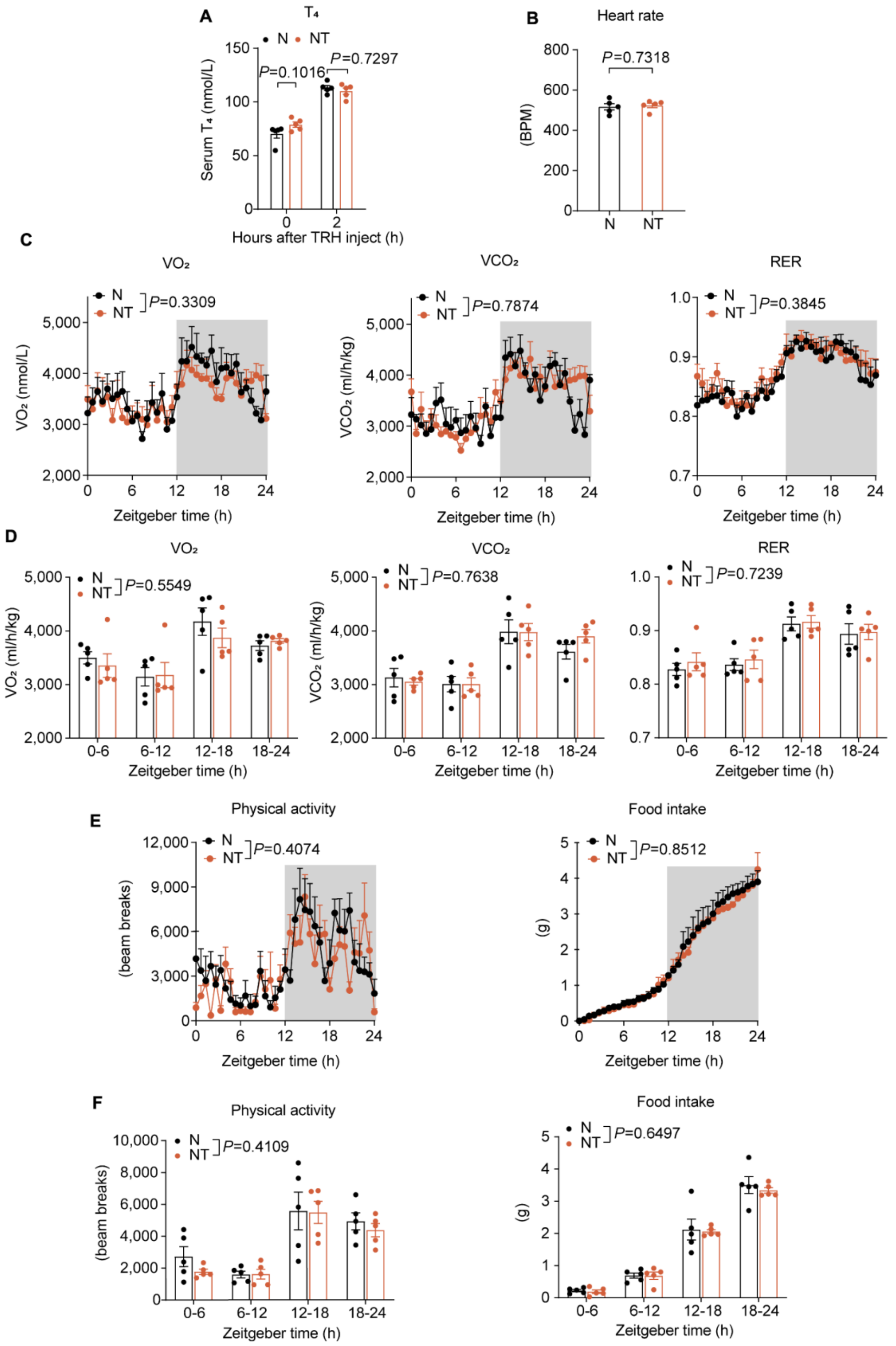

Figure S8. Physiological homeostasis of mice with a second thyroid gland; Related to Figure 5. A) Serum hormone levels in N mice and overloaded thyroid mice (NT mice) after thyrotropin-releasing hormone (TRH) stimulation. *n*=5. Serum samples were collected from mice with intra-splenic regenerated thyroid without thyroidectomy at 16 weeks post-transplantation, and T_4 concentrations were

measured by ECL. B) Heart rate measurements of NT mice with intra-splenic regenerated thyroid without thyroidectomy at 16 weeks post-transplantation, with N mice as the control. *n*=5. C,D) C)Metabolic cage tests measuring and D) statistics for VO_2, VCO_2, and RER in N mice and NT mice (mice with additional intra-splenic thyroid. *n*=5. E,F) E)Metabolic cage tests measuring and F) statistics for physical activity and food intake in the mice. *n*=5. NT mice were intra-splenic transplantation thyroid without thyroidectomy at 16 weeks post-transplantation. *n*=5 Data are presented as means ± SEM. Statistical analyses were performed using *Student's* t-test (B, C, E) and two-way ANOVA followed by *Tukey's* multiple comparisons tests for (A, D, F). NT refers to normal mice with an additional intra-splenic thyroid; ANOVA; analysis of variance.

Table S1. Blood routine examination of normal and ST mice.

Parameters	N	ST
Lymphocytes (%)	60.22±9.98	69.12±4.12
Monocytes (%)	3.54±1.22	3.56±0.62
Neutrophils (%)	36.24±8.80	27.32±4.17
Erythrocytes (10^{12}/L)	9.64±0.57	10.22±0.63
Hemoglobin (g/L)	158.80±8.44	164.80±8.90
Hematocrit (%)	51.30±3.16	52.40±7.07
Mean corpuscular volume (fL)	53.30±1.57	51.14±3.95
Mean corpuscular hemoglobin (pg)	16.44±0.65	16.10±0.12
Mean rcorpuscular hemoglobin concrntration (g/L)	309.60±12.82	317.20±26.90
Coefficient of variation of red blood cell distribution width (%)	19.36±2.28	19.36±1.34
Platelet count (10^9/L)	1,160.60±228.73	1,272.40±211.43
Mean platelet volume (fL)	5.94±0.55	5.52±0.33
Platelet distribution widths	16.48±0.50	16.16±0.48
Plateletcrit (%)	0.59±0.06	0.60±0.00

Data are presented as means ± SEM.

Table S2. Primer sequences used for RT-qPCR analysis in this study.

	Gene	Forward Primer (5'-3')	Reverse Primer (5' -3')
Thyroid	*GAPDH*	AGGTCGGTGTGAACGGATTTG	TGTAGACCATGTAGTTGAGGTCA
	NKX2-1	CGCCTTACCAGGACACCAT	CCCATGCCACTCATATTCAT
	PAX8	CAGCCTGCTGAGTTCTCCAT	CTGTCTCAGGCCAAGTCCTC
	Tg	AGGACCCGTGTGGTAGG	CTGACCCAGAGAATGGCAGT
	TPO	ACAGTCACAGTTCTCCACGGATG	ATCTCTATTGTTGCACGCCCC
	TSHR	GTCTGCCCAATATTTCCAGGATCTA	GCTCTGTCAAGGCATCAGGGT
Heart	*Myh6*	GCCCAGTACCTCCGAAAGTC	GCCTTAACATACTCCTCCTTGTC
	Esm1	GGCTGAAGTGTCACTTTTACAG	CAAGTCTCTTTGCATTCCATCC
	Aplnr	TTACTTCTTCATTGCCCAAACC	CACTACAAGCACCACGATAATG
Liver	*THRSP*	ATGCAAGTGCTAACGAAACGC	CCTGCCATTCCTCCCTTGG
	Dio 1	GCTGAAGCGGCTTGTGATATT	GTTGTCAGGGGCGAATCGG
	ME1	GTCGTGCATCTCTCACAGAAG	TGAGGGCAGTTGGTTTTATCTTT
	Per3	GCAAGCAAACAGTGACTTTTTC	TTCCAGACAGAAACGAAAACAC
	Ciart	ACCAACTCCACTGTCTCCTGTC	TCTGGGTGTCCTTTAGTCATCTCTC
	Trib3	GGAACCTTCAGAGCGACTTGTG	CGGTCAGACTGTGGAGGAGAG
	Gclc	CTATCTGCCCAATTGTTATGGC	CCTCCCGTGTTCTATCATCTAC
	Cyp2c69	TGTAGTCTTGGTGCTTTGTCTG	ACAATAGGCTGTGAGCCAAAATA
	Cyp2c40	GGCTCACAGCCTATTGTGGTA	TCAAAAACCGGAATCCTTCCTC
	Cyp21a1	CAAGATGTGGTGGTGCTAAATT	GCCTTCCACATGAGAGAGTAAT
